# Metallomic Analysis of Vitreous Humor of the Human Eye—A Post-Mortem Multielemental Study

**DOI:** 10.3390/ijms27062527

**Published:** 2026-03-10

**Authors:** Alicja Forma, Michał Flieger, Beata Kowalska, Jolanta Flieger, Andrzej Torbicz, Jacek Bogucki, Grzegorz Teresiński, Ryszard Maciejewski, Robert Rejdak, Joanna Dolar-Szczasny, Weronika Pająk, Jacek Baj

**Affiliations:** 1 Department of Forensic Medicine, Medical University of Lublin, Jaczewskiego 8b, 20-810 Lublin, Poland; 58293@umlub.edu.pl (M.F.); grzegorz.teresinski@umlub.edu.pl (G.T.); wapajak@gmail.com (W.P.); 2 Doctoral School, Medical University of Lublin, Chodźki 7 Street, 20-093 Lublin, Poland; 3 Department of Water Supply and Wastewater Disposal, Lublin University of Technology, 20-618 Lublin, Poland; b.kowalska@pollub.pl; 4 Department of Analytical Chemistry, Medical University of Lublin, Chodźki 4A, 20-093 Lublin, Poland; jolanta.flieger@umlub.edu.pl (J.F.); andrzej.torbicz@umlub.edu.pl (A.T.); 5 Faculty of Medicine, John Paul II Catholic University of Lublin, Konstantynów 1 H, 20-708 Lublin, Poland; jacek.bogucki@kul.pl; 6 Institute of Health Sciences, John Paul II Catholic University of Lublin, Konstantynów 1 H, 20-708 Lublin, Poland; ryszard.maciejewski@kul.pl; 7 Department of General and Pediatric Ophthalmology, Medical University of Lublin, 20-079 Lublin, Poland; robert.rejdak@umlub.edu.pl (R.R.); joannadolarszczasny@umlub.edu.pl (J.D.-S.); 8 Department of Correct, Clinical, and Imaging Anatomy, Medical University of Lublin, Jaczewskiego 4, 20-090 Lublin, Poland

**Keywords:** metallomics, vitreous humor, human eye, autopsy, multielemental analysis, trace elements, toxic metals, post-mortem study, ICP-MS, micronutrients

## Abstract

The elemental composition of the vitreous humor may reflect physiological and pathological processes occurring in the eye. The objective of this study was to provide a complex multielemental analysis of human vitreous humor. Vitreous humor samples (n = 57) were collected post-mortem during autopsies. Inductively coupled plasma mass spectrometry (ICP-MS) was employed to quantify micro-, trace-, ultra-trace, and toxic elements. The study showed the occurrence of elements at the ppm (Na, K, P, Ca, Mg), ppb (Al, Rb, Zn, Fe, Sr, Cu), and ppt (Ce, La, Nd, Tb) levels. Hierarchical clustering using Ward’s method and k-means analysis revealed four distinct clusters, including two major clusters representing the baseline macro- and microelement profile characteristic for the studied population. Correlations between elements revealed statistically significant (*p* < 0.05) positive and negative correlations between elements with (I) chemical similarity Ce-La, Cs-Rb, Rb-K, Ca-P, Zn-Cu, and Cs-K; (II) a possible common environmental origin, Cd-P, and Rb-P; (III) involvement in similar biological processes as K-P; and (iv) a common geochemical origin and similar biological functions, i.e., Se-Zn. The study identified several quantitative trends in the demographic and medical characteristics of the participants. Alcohol users had significantly higher Zn concentrations than non-alcohol users; women had significantly higher Ca concentrations than men; higher BMI correlated positively with Cs and negatively with Be and Cr levels; and Cu, Sb, Cd, Se, and Ca concentrations increased with age. The presence of several toxic and potentially toxic elements was identified in the vitreous body: Al (>10 ppb); Cd, Cr, Pb, Ni, Mn; and Ba (<10 ppb); As, Hg, Sb, Tl, Bi, Be (<1 ppb). The study showed that, within a given geographic region, the accumulation profiles of toxic metals are quite homogeneous, indicating common sources of exposure.

## 1. Introduction

Metallomics is an integrative and rapidly evolving field of study that focuses on the comprehensive investigation of the total content, distribution, functional roles, and interactions of metal and metalloid species within biological organisms as well as in the environment [1]. It complements genomics, proteomics, and metabolomics by elucidating how metal ions participate in essential physiological functions such as enzymatic catalysis, oxidative balance, and cellular signaling [2]. Metallomic analysis of the biological organisms aims to elucidate how the investigated elements contribute to biochemical networks and cellular regulation, serving as essential cofactors for numerous enzymes and structural components of biomolecules. The metallome reflects the dynamic equilibrium between essential and toxic metals, which collectively influence oxidative balance, mitochondrial function, signal transduction, and gene expression [3,4,5,6,7]. Alterations in this delicate equilibrium may disrupt redox homeostasis and biomolecular integrity, thereby contributing to the initiation and progression of various pathological states. Disturbances in metal homeostasis may contribute to the onset and progression of a wide range of pathological processes, including neurodegenerative, psychiatric, metabolic, and ophthalmic diseases [8,9,10,11,12,13,14,15]. Because of the involvement of metals in numerous processes occurring in the human organism, metallomic investigations provide valuable molecular insights into both physiological and pathophysiological mechanisms that might lead to disease onset and progression.

A variety of analytical methodologies have been employed in metallomic research, including flame and graphite furnace atomic absorption spectrometry (FAAS, GFAAS), inductively coupled plasma optical emission spectrometry (ICP-OES), X-ray fluorescence (XRF), atomic absorption spectrometry (AAS), and inductively coupled plasma mass spectrometry (ICP-MS) [16,17,18,19,20,21]. Among these, ICP-MS has emerged as the technique of choice for biological applications due to its exceptional sensitivity, high precision, and capability for simultaneous multi-element analysis across a wide concentration range allowing accurate quantification of both essential and toxic elements even at ultra-trace concentrations [21,22,23,24]. Its ultra-trace detection limits and minimal sample volume requirements make it particularly advantageous for ophthalmic research, where tissue availability is limited [25].

Within the eye, metal ions are indispensable for maintaining normal physiology [26]. Elements such as zinc (Zn), copper (Cu), iron (Fe), calcium (Ca), and magnesium (Mg) play key roles in enzymatic activity, phototransduction, and antioxidant defense [27,28,29]. Conversely, the accumulation of toxic metals, including lead (Pb), cadmium (Cd), mercury (Hg), aluminum (Al), thallium (Tl), or arsenic (As), can induce oxidative damage, lipid peroxidation, and protein misfolding, leading to degeneration of ocular tissues [30,31]. Dysregulation of trace element homeostasis has been implicated in the pathogenesis of various ophthalmic diseases, including age-related macular degeneration (AMD), glaucoma, diabetic retinopathy, and cataracts [32,33,34,35,36,37,38,39]. Nevertheless, the underlying mechanisms linking metal imbalance to ocular pathology remain insufficiently investigated and characterized yet, largely due to limited elemental data derived from human ocular tissues and difficulties in obtaining human ocular samples. Thus, despite significant advances in systemic metallomics, knowledge concerning the elemental composition of ocular tissues remains highly limited.

**Figure 1:**
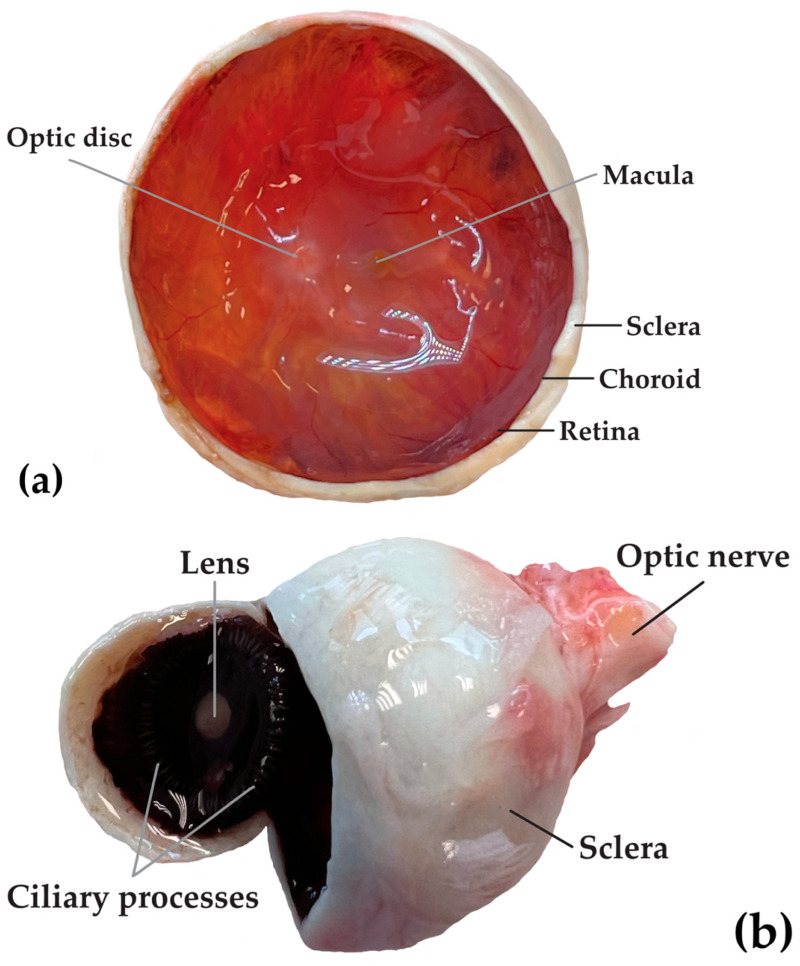
(**a**) Coronal section of the right human eye, posterior view. (**b**) The right human eyeball after anterior opening in the coronal plane. Samples were collected during an autopsy at the Department of Forensic Medicine, Medical University of Lublin, Poland.

The human eye represents a unique microenvironment in which precise ionic equilibrium is essential for maintaining transparency, intraocular pressure, and photoreceptor function [40,41,42]. Among its components, the vitreous humor, the largest intraocular component, serves as both a metabolic reservoir and a diffusion medium for nutrients, metabolites, and ions [43]. The vitreous humor, a transparent, gel-like extracellular matrix occupying approximately 80% of the eye’s volume, represents a unique biological compartment (Figure 1) [44].

It is composed primarily of water, collagen, and hyaluronic acid and functions as a diffusion medium for ions, metabolites, and signaling molecules between the retina and lens [45]. Due to its avascular nature and relative chemical stability, the vitreous humor is increasingly recognized as a valuable matrix for biochemical, toxicological, and post-mortem investigations. However, despite its diagnostic potential, comprehensive metallomic analyses of the human vitreous remain scarce. Most previous studies regarding metallomic analyses of the vitreous humor have focused on a limited number of elements or have been performed in experimental animal models, thus leaving a significant knowledge gap regarding the normative elemental composition of the human ocular environment [46,47]. Establishing normative elemental ranges and identifying potential inter-element correlations could provide important insights into the mechanisms underlying both ocular physiology and pathophysiology as well.

The present study aimed to perform a comprehensive metallomic characterization of the human vitreous humor samples obtained post-mortem. Population-based studies conducted in individuals residing within the same geographical region enable the assessment of environmental exposure risks. Markers of such environmental hazards may include the levels of toxic elements detected in biological samples. The presence of toxic elements is known to disrupt the homeostasis of other elements, particularly essential trace elements and macroelements. Therefore, such studies are crucial for evaluating the biological consequences of environmental toxin accumulation, including heavy metals and potentially toxic elements.

Previous studies investigating changes in micro- and macroelement concentrations within ocular tissues and the visual pathway have largely been conducted using animal models. When it comes to human-based studies, analyses have typically been limited to selected elements, predominantly essential elements such as sodium (Na), potassium (K), calcium (Ca), magnesium (Mg), copper (Cu), zinc (Zn), selenium (Se), and iron (Fe) [48,49]. Other research has focused on heavy metals; however, these studies were also limited in scope, most often examining only a small number of elements simultaneously, for example, lead (Pb), cadmium (Cd), mercury (Hg), and thallium (Tl) only [50]. To date, no study has simultaneously analyzed such a broad panel of elements as in our study, encompassing both essential trace elements and toxic elements, including heavy metals, in a single study. The available human studies have additionally been limited by relatively small sample size, for example, investigating only 16 or 27 individuals in one study [48,50]. Consequently, the vitreous humor remains one of the less frequently investigated human tissues with respect to elemental composition, with only a limited number of studies reported to date. The present study addresses this knowledge gap by providing a comprehensive elemental characterization of this tissue.

Using ICP-MS, we quantified the concentrations of micro-, trace-, ultra-trace, and toxic elements in vitreous samples collected from 57 deceased individuals during routine forensic autopsies at the Department of Forensic Medicine, Medical University of Lublin, Poland. Regarding the classification of the investigated elements, the biological classification which is based on the physiological role in the body classify elements into macro elements, constituting the structure of the bulk of cells and tissues (O, C, H, N, Ca, P, K, Na, S, Cl, Mg), essential (vital) trace elements Fe, I, Cu, Zn, Co, Cr, Mo, Se, Mn being the specific elements in structure of enzymes and other biologically active molecules, and toxic elements (Al, Cd, Pb, Hg, Be, Ba, Bi, Tl) and potentially toxic Ag, Au, In, Ge, Rb, Ti, Te, U, W, Sn, Zr, etc. In turn, classification of chemical elements into groups, depending on their contents in the human organism divide elements into: macro elements (O, C, H, N, Ca, P, K, Na, S, Cl, Mg) whose concentration in the body exceeds 0.01%, trace elements (Fe, Zn, F, Sr, Mo, Cu, Br, Si, Cs, I, Mn, Al, Pb, Cd, B, Rb) with concentrations ranging from 0.00001% to 0.01%, and ultratrace elements (Se, Co, V, Cr, As, Ni, Li, Ba, Ti, Ag, Sn, Be, Ga, Ge, Hg, Sc, Zr, Bi, Sb, U, Th, Rh.) with concentrations lower than 0.000001% [51,52,53,54].

The research hypothesis assumes that the accumulation of elements within the vitreous humor demonstrates variability across trace, ultra-trace, and potentially toxic elements, as well as heavy metals, although the overall elemental profile remains relatively homogeneous within a given geographic region. To address this objective, we quantified the accumulation of 51 elements in human vitreous humor, including trace, ultratrace, toxic elements, and heavy metals, while also examining interelemental relationships among the analyzed elements. To assess the homogeneity of the study group, selected statistical approaches were applied, including cluster analysis and principal component analysis (PCA). Furthermore, trends in elemental accumulation in the vitreous humor were evaluated in relation to selected variables such as age, sex, BMI, and alcohol consumption.

The objectives of the study were to (I) determine the baseline elemental composition of the human vitreous humor; (II) assess the relationships between biologically essential and toxic metals; and (III) evaluate the potential of the vitreous as a stable matrix for post-mortem metallomic studies. By providing fundamental reference data, this work contributes to the understanding of metal homeostasis in the human eye and lays the groundwork for future investigations into its alterations in ophthalmic diseases.

## 2. Results

### 2.1. Descriptive Statistics of ICP-MS Measurements for the Whole Population

Elemental distributions were summarized using the total number, minimum, maximum, median, first quartile (Q1), third quartile (Q3), mean, standard deviation, skewness, and kurtosis for each analyte. Because vitreous humor elemental profiles typically show pronounced right-skewness and occasional extreme values (as reflected by skewness/kurtosis across multiple elements), inferential analyses were interpreted with emphasis on robust (rank-based) associations and distribution-aware comparisons (Supplementary S1).

Descriptive statistics revealed distinct distributional characteristics for macroelements compared with trace and ultra-trace elements. Notably, macroelements—including sodium (Na) (−1.6), potassium (K) (−0.1), calcium (Ca) (−0.2), magnesium (Mg) (−0.2), and phosphorus (P) (+0.6)—exhibited pronounced skewness values, indicating a strong concentration of observations around central tendency measures with limited dispersion occurring before median and mean value. Additionally, macroelements were the only group of elements displaying negative skewness, reflecting a predominance of higher concentration values and relatively infrequent low-level observations.

The most abundant elements found in the vitreous humor were primarily sodium (Na) (2830.5506 ± 488.2478) [ppm], potassium (K) (569.1637 ± 171.5943) [ppm], phosphorus (P) (88.3079 ± 49.2908) [ppm], calcium (Ca) (52.6585 ± 20.6555) [ppm], and magnesium (Mg) (21.5074 ± 5.8473). Elements with concentrations >10 ppb included also aluminum (Al) (907.8153 ± 5687.1650), rubidium (Rb) (782.2083 ± 327.2737), zinc (Zn) (612.9806 ± 724.9021), iron (Fe) (320.2954 ± 379.2349), strontium (Sr) (52.6064 ± 49.0760), and copper (Cu) (37.6494 ± 42.2904). Levels <10 ppb were mainly represented by trace elements including titanium (Ti) (8.0391 ± 30.2924), manganese (Mn) (7.3572 ± 24.5612), cadmium (Cd) (5.6273 ± 8.5622), chromium (Cr) (3.0863 ± 9.1573), caesium (Cs) (2.6493 ± 1.7353), molybdenum (Mo) (2.1592 ± 3.2638), barium (Ba) (1.9414 ± 5.0468), lead (Pb) (1.4194 ± 4.1022), and nickel (Ni) (1.2349 ± 3.7422) as well as ultra-trace elements—arsenic (As) (0.2554 ± 1.1708), gallium (Ga) (0.2152 ± 0.9879), antimony (Sb) (0.1624 ± 0.2740), bismuth (Bi) (0.1360 ± 0.4266), tin (Sn) (0.1269 ± 0.3284), cobalt (Co) (0.0678 ± 0.1608), zirconium (Zr) (0.0589 ± 0.1828), palladium (Pd) (0.0172 ± 0.0210), platinum (Pt) (0.0075 ± 0.0126), thallium (Tl) (0.0050 ± 0.0142), thorium (Th) (0.0046 ± 0.0175), hafnium (Hf) (0.0029 ± 0.0069), beryllium (Be) (0.0020 ± 0.0115), and silver (Ag) (0.0005 ± 0.0039). In the whole group of elements studied in the vitreous humor, the lowest concentrations were represented by the rare earth metals, namely cerium (Ce) (0.3313 ± 0.5274), lanthanum (La) (0.0735 ± 0.1299), neodymium (Nd) (0.0240 ± 0.0603), samarium (Sm) (0.0178 ± 0.0338), praseodymium (Pr) (0.0118 ± 0.0218), erbium (Er) (0.0085 ± 0.0164), dysprosium (Dy) (0.0066 ± 0.0136), gadolinium (Gd) (0.0047 ± 0.0105), europium (Eu) (0.0040 ± 0.0083), holmium (Ho) (0.0027 ± 0.0049), and terbium (Tb) (0.0021 ± 0.0043). All of the abovementioned rare earth metal concentrations are presented in ppb.

### 2.2. Inter-Element Relationships in Vitreous Humor

**Figure 2:**
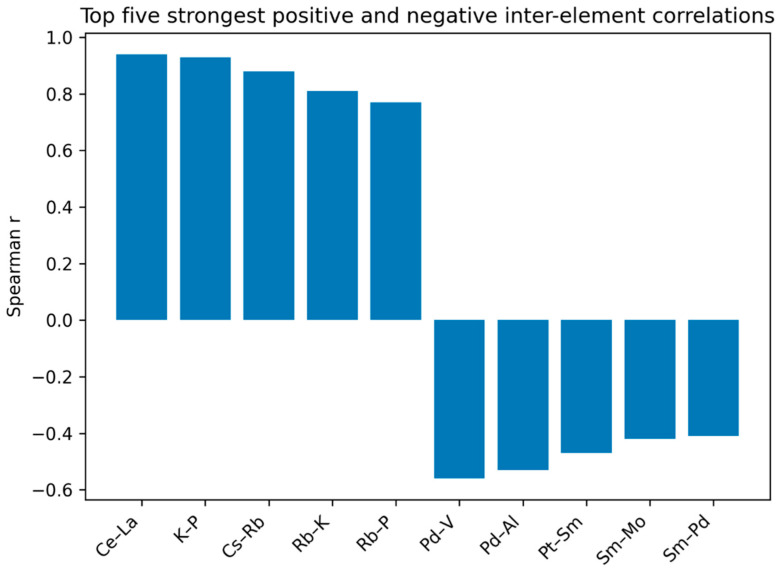
Summary table reporting the five strongest positive and five strongest negative inter-element correlations, together with their correlation coefficients.

The results of the correlation analysis between the concentrations of the analyzed elements [ppb] are presented as a heatmap of the lower triangle of the correlation coefficient matrix (Supplementary S2). Below, we presented the five strongest positive and the five strongest negative inter-element correlations (Figure 2).

The analysis demonstrated that the strongest positive correlations (r > 0.80) were observed between cerium and lanthanum (Ce–La; r = 0.94), potassium and phosphorus (K–P; r = 0.93), cesium and rubidium (Cs–Rb; r = 0.88), and rubidium and potassium (Rb–K; r = 0.81), The other strong positive correlations (r ≥ 0.60) were observed between rubidium and phosphorus (Rb-P; r = 0.77), cadmium and phosphorus (Cd-P; r = 0.76), cesium and potassium (Cs-K; r = 0.75), zinc and copper (Zn-Cu; r = 0.71), cesium and phosphorus (Cs-P; r = 0.70), calcium and phosphorus (Ca-P; r = 0.67), copper and phosphorus (Cu-P; r = 0.67), phosphorus and magnesium (P-Mg; r = 0.67), selenium and zinc (Se-Zn; r = 0.66), cadmium and potassium (Cd-K; r = 0.66), selenium and copper (Se-Cu; r = 0.64), molybdenum and zinc (Mo-Zn; r = 0.64), gadolinium and lanthanum (Gd-La; r = 0.64), bismuth and tin (Bi-Sn; r = 0.64), selenium and phosphorus (Se-P; r = 0.63), palladium and molybdenum (Pd-Mo; r = 0.62), cadmium and copper (Cd-Cu; r = 0,62), uranium and thorium (U-Th; r = 0.61), gadolinium and cerium (Gd-Ce; r = 0.61), barium and cobalt (Ba-Co; r = 0.60), and cerium and cobalt (Ce-Co; r = 0.60).

In contrast, the strongest negative correlations (r < −0.40) were identified between palladium and aluminum (Pd–Al; r = −0.53), palladium and vanadium (Pd–V; r = −0.56), samarium and molybdenum (Sm–Mo; r = −0.42), samarium and palladium (Sm–Pd; r = −0.41), and platinum and samarium (Pt–Sm; r = −0.47).

### 2.3. Hierarchical Clustering Using Ward’s Method

**Figure 3:**
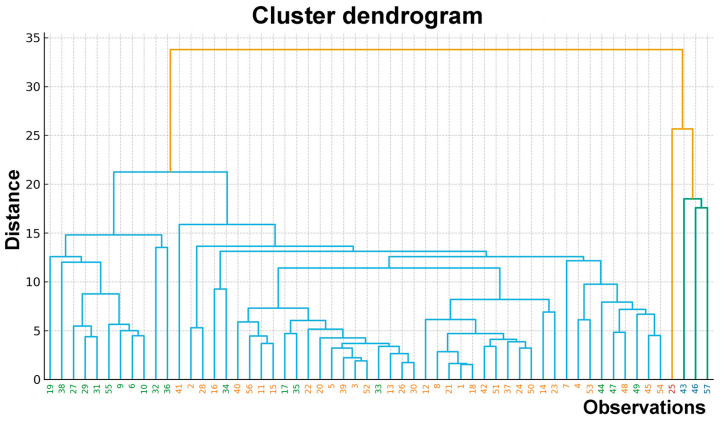
Hierarchical clustering dendrogram illustrating similarities among individual vitreous humor samples based on elemental concentrations. Each label on the *x*-axis represents a single individual (n = 57). The *y*-axis indicates the linkage distance between clusters calculated using Euclidean distance, and hierarchical clustering was performed using Ward’s linkage method. Branch colors denote distinct clusters identified at the selected threshold: yellow indicates cluster 2 (n = 35), the largest background cluster; green represents cluster 3 (n = 18), characterized by elevated macroelements and selected microelements; blue corresponds to cluster 1 (n = 3), showing strong enrichment in trace elements and rare earth elements; and red represents cluster 4 (n = 1), representing an extreme outlier case.

Hierarchical cluster analysis was performed using Ward’s linkage method based on Euclidean distances calculated from standardized elemental concentrations. Inspection of the Ward hierarchical dendrogram revealed a well-defined multilevel structure of similarity among vitreous humor samples, reflecting coordinated multi-elemental concentration patterns. At lower linkage distances, samples clustered into several compact subgroups characterized by relatively homogenous elemental profiles, whereas at higher aggregation levels, a marked increase in linkage distance was observed, indicating the presence of chemically distinct subpopulations within the cohort (Figure 3).

Ward’s method was selected due to its tendency to form compact and internally homogeneous clusters while minimizing within-cluster variance. By analyzing the Euclidean distances between linkages on the dendrogram, two main branches can be distinguished: a dominant branch encompassing the majority of cases characterized by an elemental composition close to the overall mean, and a second, less numerous branch grouping cases enriched in macroelements and selected trace elements. The dendrogram also includes a small number of observations forming peripheral branches that merge with the main structure only at high distance levels, which is likely related to extreme or atypical metallomic signatures. Thus, the dendrogram topology supports a four-cluster solution, comprising two dominant clusters representing most of the studied population (clusters 2 and 3), as well as two smaller clusters (clusters 1 and 4) characterized by distinctly different elemental compositions. The latter occupied isolated positions within the dendrogram, supporting their interpretation as chemically specific subgroups rather than gradual extensions of the main distribution. The hierarchical structure illustrated graphically in Figure 3 may serve as a basis for further classification using the k-means method and for detailed profiling of clusters in terms of elemental composition. The agreement between dendrogram-based grouping and centroid-based partitioning further confirms the stability of the identified metallomic subtypes and suggests that the observed sample stratification reflects persistent multidimensional chemical differences rather than artifacts of a single clustering algorithm.

Inspection of the dendrogram revealed a clear hierarchical structure with a pronounced increase in linkage distance at higher levels of aggregation, indicating the presence of distinct chemical groupings among the vitreous humor samples. Based on the dendrogram topology and linkage distances, a four-cluster solution was considered optimal and was further explored in subsequent analyses. Based on hierarchical clustering analysis using Ward’s method, four clusters were identified: cluster 1 (n = 3), cluster 2 (n = 35), cluster 3 (n = 18), and cluster 4 (n = 1). Clusters 2 and 3 are the most representative, comprising the majority of the studied population. In contrast, clusters 1 and 4 included only a small number of cases and were characterized by markedly distinct mineral composition of the analyzed tissues compared with the remaining clusters.

The cluster analysis yielded a somewhat atypical distribution due to unequal cluster sizes. Two clusters (cluster 2 and 3) fulfilled the formal methodological criteria and reached a representative size; therefore, they were considered the primary and most reliable structures for further interpretation. These clusters met the key assumptions of cluster analysis, particularly regarding adequate and representative sample numbers, and thus constituted the main basis for the analytical conclusions. In addition, two smaller and non-representative clusters (cluster 1 and 4) were identified. Owing to their limited size, they did not meet the criteria for representative clusters and were interpreted as outlier groups. These clusters are presented only for illustrative purposes and should be regarded as exploratory findings rather than statistically robust structures.

#### Cluster Delineation and Integration with k-Means Classification

**Figure 4:**
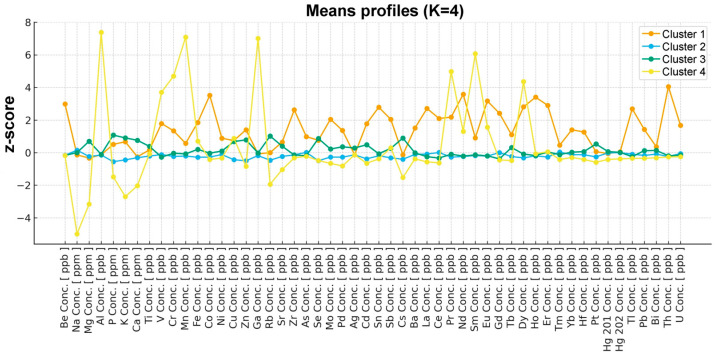
Mean standardized (z-score) profiles of elemental concentrations across clusters identified by Ward’s hierarchical clustering (k = 4). Each line represents the cluster-specific average z-score for individual elements, illustrating distinct elemental accumulation patterns among clusters.

To refine cluster assignment, hierarchical clustering results were complemented by k-means clustering with K = 4. The selected K value was derived from the hierarchical solution. Inspection of the ward dendrogram revealed a pronounced increase in linkage distances at higher fusion levels, indicating that further merging would combine chemically dissimilar samples. Accordingly, a four-cluster partition was chosen as the most parsimonious and chemically interpretable cut of the dendrogram and was subsequently adopted in k-means to refine cluster membership and facilitate downstream profiling. Cluster membership was superimposed on the Ward dendrogram using color-coded labels (Figure 4), allowing direct comparison between both clustering approaches.

The combined analysis confirmed a high level of concordance between Ward’s hierarchical structure and k-means partitioning, supporting the robustness of the four-cluster solution. Most samples grouped consistently across both methods, with discrepancies limited to borderline cases located near cluster boundaries in the dendrogram.

The four identified clusters differed markedly in terms of their standardized elemental concentration profiles, as illustrated in the mean z-score profiles (Figure 4).

K-means clustering (k = 4) was based on 52 standardized variables (elemental concentrations), and analysis revealed four major clusters.

Cluster 2 (n = 35) constitutes a ‘background’/near-average profile. It is the largest cluster, characterized by centroids very close to zero for the majority of elements. It is distinguished by slightly lower values for several elements, including phosphorus (P), potassium (K), magnesium (Mg), zinc (Zn), copper (Cu), selenium (Se), rubidium (Rb), caesium (Cs), and cadmium (Cd) (mean approximately −0.4 to −0.6 z-score). This cluster can be regarded as a baseline chemical profile, representing samples with moderately reduced concentrations of selected macro- and microelements, without pronounced extremes.

Cluster 3 (n = 18) was characterized by elevated macroelements and selected microelements. Cluster 3 shows clearly elevated concentrations of macroelements such as phosphorus (P), potassium (K), calcium (Ca), and magnesium (Mg), as well as microelements such as zinc (Zn), copper (Cu), selenium (Se), rubidium (Rb), and caesium (Cs). For most of these elements, values reach approximately +0.7 to +1.1 z-score. Thus, this cluster represents chemically enriched samples compared to the overall mean, particularly with respect to macroelements and several trace elements.

Cluster 1 (n = 3) presented strong enrichment in trace elements and rare earth elements. This small cluster exhibits very high concentrations (approximately 2-4 z-scores) for numerous trace metals and rare earth elements including thorium (Th), neodymium (Nd), cobalt (Co), holmium (Ho), europium (Eu), beryllium (Be), erbium (Er), dysprosium (Dy), lanthanum (La), tin (Sn) and other rare earth elements. In contrast, macroelements (sodium (Na), magnesium (Mg), calcium (Ca)) show values slightly below the mean. This indicates a distinctly ‘trace-element-dominated’ chemical signature, in which the samples are not enriched in major cations but display marked accumulation of multiple trace and rare earth elements.

Cluster 4 (n = 1) presented a single extreme outlier case. A single sample forms an independent cluster with a highly extreme elemental profile, characterized by very high concentrations of aluminum (Al), manganese (Mn), gadolinium (Ga), samarium (Sm), praseodymium (Pr), chromium (Cr), dysprosium (Dy), and vanadium (V) (approximately 4-7 z-scores). This cluster simultaneously presents very low concentrations of sodium (Na), magnesium (Mg), potassium (K), calcium (Ca), rubidium (Rb), caesium (Cs), phosphorus (P), and strontium (Sr) (approximately −1.5 to −5 z-scores). This represents a classical chemical outlier, markedly different from all other samples. It should be emphasized that this cluster has an outlier character and should be interpreted with caution, more as an extreme individual observation rather than as a representative chemical subtype of vitreous body tissue. It should be noted that this sample was obtained for a homeless person, without any medical documentation and additional patient history, because of the lack of the possibility to obtain such information. The subject was a male who died because of a sudden death; there is no information about his age. Medical information of the subject revealed during the autopsy included information about the atherosclerosis of the coronary arteries and aorta, as well as hepatic steatosis. The patient presented a BMI classified as normal (BMI = 23.67), and no alcohol was detected from the blood samples obtained during the autopsy, suggesting that when the patient died, he was not intoxicated with alcohol.

Overall, the clustering analysis reveals a high degree of chemical homogeneity across most samples, with cluster separation driven primarily by quantitative differences in macroelement concentrations, and to a lesser extent, coordinated enrichment of selected trace elements. The presence of a single extreme outlier highlights the potential for rare, atypical elemental accumulation patterns in vitreous humor samples. Nevertheless, regarding the two outlier clusters (cluster 1 and 4), it should be noted that since they did not reach the representative size, they were presented for illustrative purposes only, and the results of this analysis should be interpreted with considerable caution.

### 2.4. Principal Component Analysis (PCA) of Vitreous Humor Elemental Patterns

#### 2.4.1. Suitability of PCA

PCA was used to identify latent multi-element accumulation patterns in vitreous humor. Suitability of the correlation structure for PCA was confirmed by Bartlett’s test of sphericity performed on the 52 × 52 correlation matrix (χ^2^(1326) = 4147.84; *p* < 0.001), demonstrating that the correlation matrix significantly differed from the identity matrix and that the dataset contained substantial shared variance (*p* < 0.001) (Supplementary S3).

#### 2.4.2. Component Retention and Explained Variance

Using the Kaiser criterion (λ > 1), thirteen principal components were retained (PC1–PC13), jointly explaining (86.34%) of the total variance (Table 1).

The leading components accounted for the largest variance proportions: (PC1: 23.88%), (PC2: 14.83%), (PC3: 8.91%), and (PC4: 7.21%). The scree plot showed a pronounced “elbow” with steep declines for (PC1–PC4) followed by progressively smaller variance increments for later components, supporting a parsimonious focus on the first components for visualization and interpretation (PC1–PC4) (Supplementary S4).

#### 2.4.3. Interpretation of Major Components

PCA performed on 52 variables identified 13 components with eigenvalues greater than 1 (Kaiser criterion). Together, these components explain 86.34% of the total variance. The first principal component (PC1) explains 23.88% of the variance, the second 14.83%, the third 8.91%, and the fourth 7.21%. Beyond the fourth component, the incremental gain in explained variance decreases markedly, as illustrated by the scree plot.

High communality values (often exceeding 90%) indicate that the 13 retained components provide a very good reconstruction of the variance of the original variables. Thus, the majority of information on the chemical variability of the samples is captured within the principal component space.

Components were used to interpret dominant accumulation signatures in vitreous humor. PC1 (23.9% variance) was primarily driven by high positive contributions of (Co, Th, Ho, Eu, Er, Sn, Nd, Be, Dy, Sb), consistent with a trace-element-rich signature. The concentrations of these elements are at the level of detection corresponding to hundredths to thousandths of a ppb. PC2 (14.8% variance) contrasted negative loadings of macroelements and related ions (K, P, Ca, Mg, Rb, Cs) against positive loadings of (Mn, Al, Sm, Ga), highlighting a compositional axis separating macroelement-rich profiles from specific trace-metal enrichment (Table 2).

### 2.5. Trends in Element Concentration Levels

For all investigated variables, namely sex, age, alcohol consumption, and BMI, all 51 elements and their potential associations were analyzed for each variable. Amongst all studied elements with the abovementioned variables, none of the observed trends reached statistical significance. However, trends approaching statistical significance were observed only for a limited number of elements, and only these relationships are presented in the following paragraph. Accordingly, only associations and trends close to statistical significance are presented, included in the graphical presentation, and these are further discussed.

#### 2.5.1. Age and Elemental Concentration

The analysis revealed six statistically significant correlations (five positive and one negative) between elemental measures and age. Older age was associated with higher values for Ca [ppm], Cu [ppb], Se [ppb], Cd [ppb], and Sb [ppb], while lower values were observed for V [ppb]. All correlation coefficients were statistically significant (*p* < 0.05). The remaining correlations were not statistically significant (Table 3).

**Figure 5:**
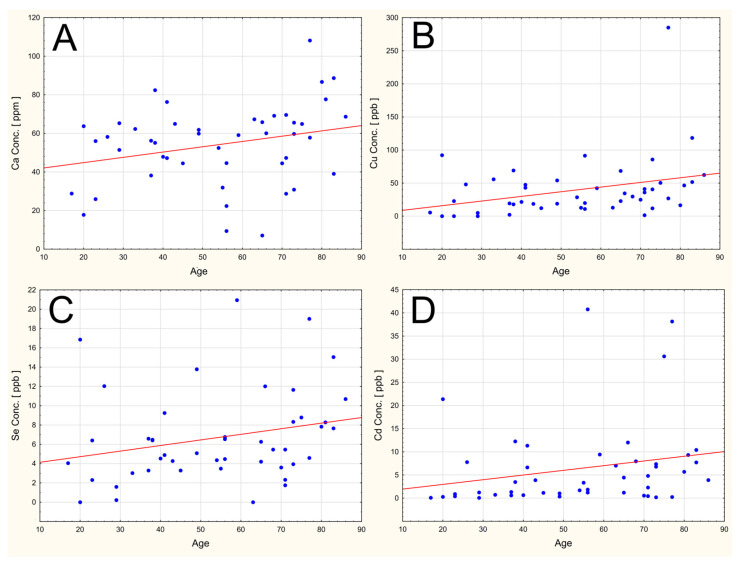
Scatter plots for age-related trends in the concentrations for: (**A**) calcium (Ca), (**B**) copper (Cu), (**C**) selenium (Se), and (**D**) cadmium (Cd).

Figure 5 provides a graphical representation of selected age-related trends in elemental concentrations that were identified in the correlation analysis (Figure 5).

#### 2.5.2. BMI and Elemental Measures

**Figure 6:**
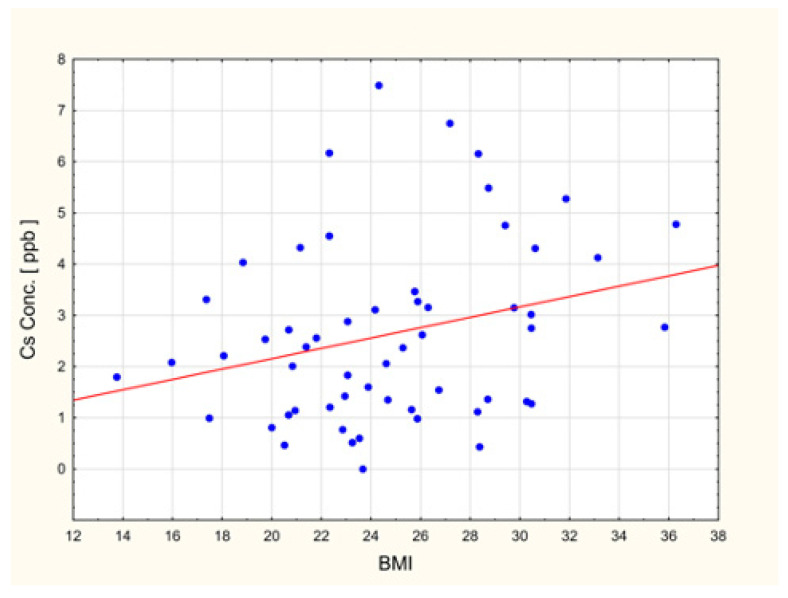
Scatter plot for BMI-related trends in the concentrations for caesium (Cs).

**Figure 7:**
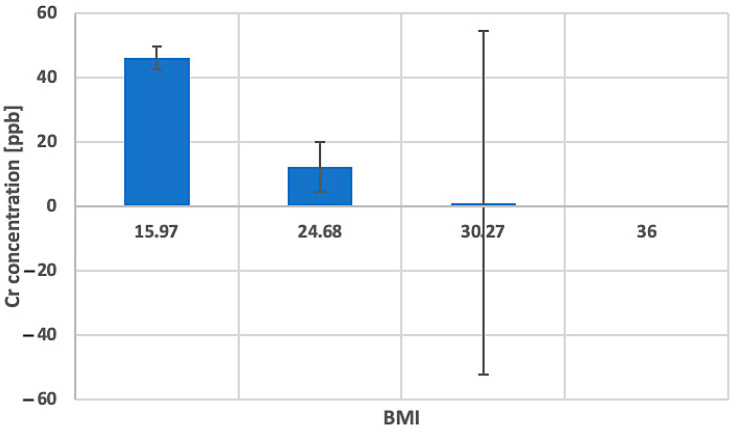
Bar graph representing changes in Cr concentration [ppb] for different BMI values with RSD error bars.

The analysis identified three statistically significant correlations (including one positive and two negative correlations) between elemental measures and body mass index (BMI). Increasing BMI was associated with a higher Cs [ppb], whereas lower values were observed for Be [ppb] and Cr [ppb]. All correlation coefficients were statistically significant (*p* < 0.05). The remaining correlations were not statistically significant (Table 4) (Figure 6 and Figure 7).

#### 2.5.3. Sex-Related Differences

**Figure 8:**
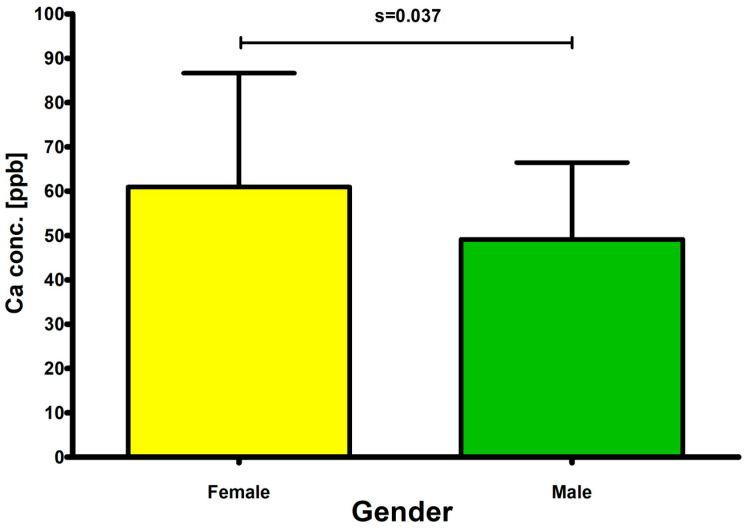
Comparison of calcium (Ca) concentrations (ppb) between female and male subjects.

Sex differences in elemental measures were evaluated, and one variable showed a statistically significant difference between women and men (*p* < 0.05). Females exhibited significantly higher values for calcium (Ca) [ppm] (*p* = 0.037) in comparison to males (Table 5) (Figure 8).

#### 2.5.4. Alcohol-Related Differences

**Figure 9:**
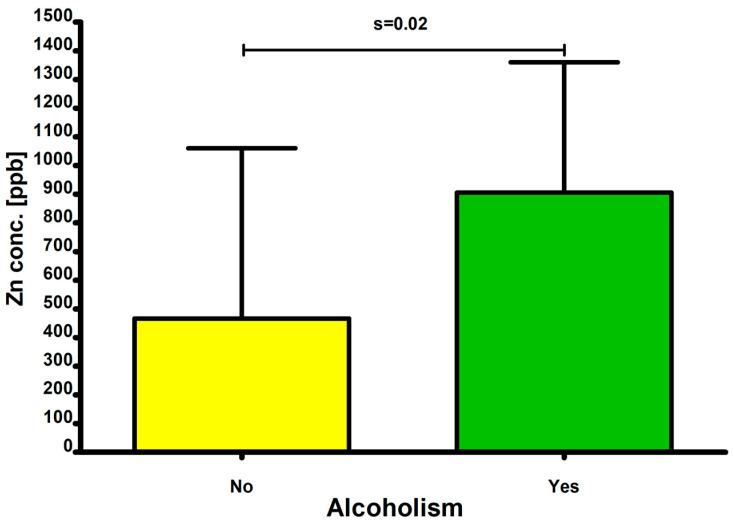
Comparison of zinc (Zn) concentrations (ppb) between individuals with and without a history of alcoholism.

Analysis of statistically significant differences in elemental concentrations between subjects affected by alcoholism and those not affected by alcoholism was performed. Zn [ppb] (*p* = 0.02) was significantly higher in subjects affected by alcoholism (Figure 9).

The remaining differences were not statistically significant (Table 6).

## 3. Discussion

Numerous studies have reported significant alterations in elemental concentrations in tissues of patients with various diseases. According to current knowledge, disturbances in microelement levels within the organ of vision might play an important role in the pathogenesis of ophthalmic diseases such as glaucoma, cataract, AMD, or diabetic retinopathy [39,55,56,57].

The first study investigating the presence of heavy metals in ocular tissue was conducted by Zeimer et al. [58]. Subsequently, Erie et al. [50] performed post-mortem analysis of various tissues and fluids of the human eye, including aqueous and vitreous humor using ICP-MS to determine elemental composition. In 2014 [59], TXRF analysis of the lens and aqueous humor in living patients with cataracts revealed higher levels of chromium (Cr) and manganese (Mn) in both matrices, elevated barium (Ba) concentrations in the lens, and increased nickel (Ni) levels in aqueous humor.

So far, most studies have focused on iron (Fe), zinc (Zn), copper (Cu), selenium (Se), and chromium (Cr). The selection of these particular elements is driven by their involvement in combating oxidative stress induced by reactive oxygen species (ROS) due to their substantial redox potential. Selenium (Se), for example, as a key component of glutathione peroxidase, counteracts oxidative damage in the lens and retina, similarly to zinc (Zn) [60,61,62]. Therefore, elemental analysis of ocular tissues may represent a useful tool enabling identification of the causes of multiple dysfunctions of the organ of vision.

It is well established that electrolyte balance is crucial, for instance, for maintaining lens transparency. Inorganic ions are transported through ion channels such as potassium (K), sodium (Na), chloride (Cl), and calcium (Ca) channels via ion exchange mechanisms (Na^+^/H^+^, Na^+^/Ca^2+^, HCO_3_^−^/Cl^−^) or active transport mediated by Na^+^/K^+^-ATPase [63]. An association between abnormal sodium (Na) accumulation or impaired Na^+^/K^+^-ATPase activity and human lens opacification has been reported by the researchers [64]. Magnesium (Mg) deficiency, accompanied by ATPase dysfunction, may also contribute to the onset of cataract [58,65].

Trace elements are estimated to account for approximately 0.1% of the total content of human tissues. The World Health Organization (WHO) classifies them into three subgroups: (1) elements essential for proper physiological function, (2) elements of limited necessity, and (3) elements that are toxic to the organism [66]. Most studies addressing changes in microelement concentrations within ocular tissues and the visual pathway have been primarily conducted using animal models or have focused on selected elements only [56].

With regard to human studies, to date, only selected ocular tissues have been investigated with respect to elemental composition. So far, the majority of the reported studies focus on the lens and aqueous humor, which are typically collected during routine cataract surgery from living patients [56,57,67,68,69,70,71,72]. Studies on other tissues are possible using autopsy tissue. These tissues can be used to investigate the pathogenesis of various ophthalmic diseases and expand our understanding of the physiology of the human eye.

It has been observed that increased intake of iron (Fe), calcium (Ca), and selenium (Se) may elevate the risk of glaucoma development [73,74]; however, the concentrations that may actually induce pathological processes within the organ of vision remain unknown. Additionally, altered elemental concentrations within the vitreous humor appear to influence the pathogenesis of various glaucoma subtypes [75]. In patients with glaucoma, increased concentrations of iron (Fe) and nickel (Ni), along with decreased levels of chromium (Cr), aluminum (Al), and manganese (Mn), have been reported in lenses compared with controls obtained from individuals without diagnosed glaucoma [76]. Furthermore, accumulation of toxic elements within the anterior chamber has been observed in glaucoma patients [39]. Fluctuations in elemental concentrations within the aqueous humor may also contribute to the onset of cytomegalovirus retinitis [57]. Increased amounts of iron (Fe) and copper (Cu) within the organ of vision have also been reported in cases of drusen [77].

The vitreous humor represents a unique and stable biological matrix for post-mortem biochemical and elemental investigations [78,79,80,81]. In recent years, increasing attention has been directed toward its potential use in trace and ultra-trace elemental analysis, particularly in the context of systemic exposure, chronic accumulation, and post-mortem redistribution processes [49]. Nevertheless, the studies regarding the vitreous humor are highly limited to several papers, most probably because of the difficulties with collecting vitreous humor samples from living patients. So far, only chosen elements were investigated in the human vitreous humor samples, including sodium (Na), potassium (K), chloride (Cl), calcium (Ca), magnesium (Mg), copper (Cu), zinc (Zn), selenium (Se), and iron (Fe) [48,82,83,84]. To the best of our knowledge, our study is the first to investigate such a high number of elements, including heavy metals and elements that might be potentially toxic to humans, also being characterized by a substantially larger study cohort compared with previously published reports.

The present study expands this field by providing a comprehensive multielemental characterization of human vitreous humor, encompassing microelements, trace elements, heavy and potentially toxic metals, as well as rare earth elements. The observations made based on the results of our study are discussed in the following subparagraphs. The discussion addresses the homogeneity of the studied group in terms of elemental composition, the accumulation of toxic elements, interelemental correlations, as well as trends associated with age, BMI, alcohol consumption, and sex.

### 3.1. Cluster Homogeneity

Hierarchical cluster analysis based on Ward’s method revealed a high degree of internal homogeneity within the identified clusters, indicating that the proposed groups represent closely related metallomic profiles of the vitreous humor under physiological and postmortem conditions.

In our study, macronutrients, including sodium (Na), potassium (K), calcium (Ca), magnesium (Mg), and phosphorus (P), were found in the highest amounts in the vitreous humor. These elements are known to play a crucial role in maintaining osmotic balance, ionic gradients, and electrochemical homeostasis in ocular tissues. The observed homogeneity of the clusters, therefore, enhances the interpretability of any detected abnormalities in the context of ocular pathophysiology and their potential usefulness in understanding and treating selected ophthalmological diseases.

The observed accumulation of toxic metals, such as cadmium (Cd), arsenic (As), aluminum (Al), thallium (Tl), lead (Pb), or mercury (Hg), is noteworthy given their well-documented toxicity and potential adverse implications for human health [85,86,87,88,89,90]. Importantly, the high similarity of the clusters with respect to trace and toxic element profiles indicates that the vitreous humor does not exhibit significant heterogeneity in metal accumulation among individuals residing in the same geographic area. The established concentration ranges for over 50 elements can be used to define reference ranges in the vitreous humor.

These distributional patterns suggest tight physiological regulation of macroelement concentrations within the vitreous humor, consistent with their essential roles in maintaining ionic balance, osmotic pressure, and metabolic stability of ocular tissues. In contrast, trace and ultra-trace elements generally showed positively skewed distributions with greater kurtosis, indicating greater relative variability and more outliers with higher concentrations and a greater probability of unexpected accumulation.

Some of the observed correlations may result from similarities in ionic radius, charge, and coordination chemistry of specific element pairs, such as Ce-La, Cs-Rb, Cs-K, Rb-K, Zn-Cu, and Ca-P [72,91,92,93,94,95,96]. These physicochemical similarities enable certain elements to utilize shared transport pathways, binding sites, or storage compartments. Most of the observed negative correlations, reflecting partial compensatory accumulation of chemically related elements in response to reduced availability of their analogs, did not exceed an absolute correlation coefficient of r = 0.6, whereas stronger correlations (r > 0.6) were exclusively positive. The presence of such interelement relationships suggests that these elements may participate in overlapping biochemical or physicochemical processes and, under specific conditions, may partially substitute for each other or act synergistically. This phenomenon is commonly referred to as ionic mimicry, a well-recognized concept in bioinorganic chemistry and metal biology [97].

From a clinical perspective, the combination of high kurtosis and negative skewness observed for macroelement concentrations suggests that sporadic reductions in their concentrations relative to the mean value are more likely. Conversely, for trace and ultra-trace elements, high kurtosis and positive skewness indicate the potential for accumulation of these elements in the vitreous.

### 3.2. Toxic and Rare Earth Element Accumulation

In our study, we observed the accumulation of toxic elements in the vitreous humor of the studied individuals. The highest concentrations with levels of >10 ppb were reported for aluminum (Al), which was the most abundant toxic element in the studied samples. The concentrations <10 ppb were observed for such toxic elements as cadmium (Cd), chromium (Cr), molybdenum (Mo), lead (Pb), and nickel (Ni), while the lowest concentrations with the levels of <1 ppb were determined for arsenic (As), bismuth (Bi), antimony (Sb), tin (Sn), thallium (Tl), thorium (Tr), and mercury (Hg). The results of our study remain consistent with observations reported in the literature. Importantly, toxic and ultra-trace elements showed to accumulate in human vitreous humor presented in our study, have been previously detected in other human tissues, including blood, brain, meninges, or liver, where their biological and toxicological effects have been extensively described [9,11,13,98,99,100,101,102]. Even relatively small concentration changes within the ppb range may therefore reflect biologically meaningful exposure patterns, particularly in the context of chronic environmental accumulation.

So far, studies regarding either the cumulation of chosen toxic elements in the eyes or the general impact of environmental exposition of the chosen elements on the organ of vision including the onset/progression of ophthalmic diseases is highly limited only to chosen elements such as abovementioned cadmium (Cd) and lead (Pb), but also aluminum (Al), mercury (Hg), arsenic (As), caesium (Cs), or nickel (Ni). Cadmium (Cd) was reported to accumulate in the human body with age, primarily in the kidneys and liver, but also in bones and retina, which was proposed to be potentially linked to AMD [103,104,105,106,107]. Aluminum (Al) tends to accumulate in the human aqueous humor which can also be associated with the onset of various ophthalmic diseases, taking into consideration the high toxicity of aluminum (Al) and its negative impact on various tissues of the human organism [39,87]. Accumulation of mercury (Hg) was described to have cataractogenic potential [108]. An animal study indicated that arsenic (As) intoxication may lead to accumulation of this element primarily in the lens, which could eventually lead to excessive oxidation stress within the lens, potentially leading to lens opacification [109]. Several toxic metals, such as nickel (Ni), lead (Pb), cadmium (Cd), mercury (Hg), bismuth (Bi), aluminum (Al), and silver (Ag) were also reported to accumulate in the retinal pigment epithelium and choriocapillaries and are hypothesized to be one of the contributing factors of AMD [32]. In addition, chromium (Cr) and caesium (Cs) were identified in human cataractous lenses [110]. The ocular exposure to rare earth metals might be associated with the onset of corneal injury, conjunctivitis, or even corneal scarring and opacity [111]. However, the literature in this matter is very scarce, and it should also be noted that rare earth metals, due to their high toxicity and negative impact on human tissues, tend to accumulate rather in low doses [112,113,114,115,116]. Essential trace elements, including cobalt (Co), zinc (Zn), copper (Cu), selenium (Se), and manganese (Mn), were also investigated in terms of accumulation and environmental exposure to ocular tissues. Cobalt (Co) toxicity might present as optic neuropathy, retinopathy, and chorioretinal degeneration [117,118,119]. Optic neuropathy might also be a result of nutritional deficiency of zinc (Zn); however, it is considered to be very uncommon [120]. Copper (Cu), selenium (Se), and manganese (Mn) present significant antioxidant defense mechanisms in the human eye, and imbalances in their concentrations might contribute to the induction of oxidative stress, which might ultimately lead to the onset of glaucoma [29,55,121].

In our study, rare earth elements were detected in the studied vitreous humor samples at ultra-trace concentration levels. Rare earth metals including holmium (Ho), europium (Eu), erbium (Er), neodymium (Nd), and dysprosium (Dy) contributed substantially to the overall variability of the investigated population, as indicated by their strong loading within the first principal component (PC1, 23.9% of total variance explained), suggesting that even minimal fluctuations in their concentrations may reflect differences in environmental exposure or physicochemical behavior. The abovementioned group also includes other groups of elements, namely cobalt (Co), thorium (Th), tin (Sn), beryllium (Be), and antimony (Sb). In this group, beryllium (Be), antimony (Sb), and thorium (Th) are considered toxic elements with well-documented toxicity, being responsible for respiratory and cardiovascular issues, gastrointestinal and skin irritations, bone marrow and kidney damage, or even cancer [122,123,124]. Tin (Sn) and cobalt (Co) are reported to be potentially toxic at elevated exposure levels. Inorganic tin (Sn) is poorly absorbed by the body and presents low toxicity, while organic tin (Sn), used in pesticides, plastics, or paint, can be easily absorbed, showing high toxicity and causing severe health effects to humans [125]. Cobalt (Co) act as an important cofactor in the human organism; however, in excessive amounts and in its inorganic form, it might present significant toxicity [126]. Regarding rare earth metals, they generally accumulate in the human body at very low concentration levels, but research about the accumulation of these elements in human tissues is highly limited. In addition, rare earth metals present incompletely characterized toxicological profiles, and available research remains inconsistent on this topic [112,127,128]. Although these elements seem to be the primary drivers of variability within the population in our study, the physiological and toxicological relevance of rare earth metals in the human organism remains incomplete and warrants further research.

### 3.3. Inter-Element Correlation Structure and Chemical Co-Accumulation Patterns

The inter-element correlation analysis revealed a complex network of statistically significant relationships. Strong positive correlations were identified between potassium and phosphorus (K-P), cerium and lanthanum (Ce-La), caesium and rubidium (Cs-Rb), or rubidium and potassium (Rb-K). Potassium (K) and phosphorus (P) present an interconnected relationship, with the kidney being a primary organ responsible for the proper homeostasis of the abovementioned elements [129]. Cerium (Ce) and lanthanum (La) are adjacent elements in the periodic table belonging to the lanthanide series, presenting many similar chemical and physical properties. It was observed that these two elements present a very similar mode of action at the metabolic level as well as similar biological pathways [130]. From a medical perspective, such correlation structures suggest that elemental accumulation in vitreous humor is not random but governed by physicochemical properties, systemic circulation patterns, and possibly barrier-specific transport processes. This has important implications for interpreting elemental data in forensic and clinical contexts, particularly when assessing combined exposures or mixed toxicological profiles.

### 3.4. Trends for Elemental Composition of Vitreous Humor with Age, BMI, Alcohol Consumption, and Sex

#### 3.4.1. Age

Several statistically significant correlations between age and selected elemental measures have been reported in our study. With increasing age, the concentrations of physiologically important elements such as copper (Cu), selenium (Se), and calcium (Ca), as well as the toxic element cadmium (Cd), were observed to increase. Conversely, negative correlations were found for vanadium (V).

The observed relationship between age and cadmium (Cd) concentration is particularly noteworthy. Cadmium (Cd) is a well-known toxic metal with an exceptionally long biological half-life, typically estimated at 10 to 30 years. It accumulates primarily in the kidneys and liver, but also in the lungs, prostate, and pancreas [131]. Accumulation of cadmium (Cd) in the vitreous humor was already reported in the 1990s [132] and likely reflects chronic exposure throughout life rather than acute poisoning. Cadmium (Cd), as well as lead (Pb), has been noted to accumulate particularly in the retinal pigment epithelium and choroid [133]. Exposure to cadmium (Cd) is associated with an increased risk of eye diseases, including cataracts and glaucoma, while environmental exposure to lead (Pb) increases the risk of cataracts and macular degeneration [134]. Our findings confirm previous reports that the vitreous humor is a site of chronic Cd accumulation. Regarding copper (Cu) accumulation, this element concentrations were reported to generally increase in various human tissues such as plasma, liver, or brain as part of the normal aging process [135,136]. This is primarily because reduced antioxidant enzymes function as well as an imbalance in copper (Cu) regulation leading to oxidative stress and cuproptosis [137]. With respect to age and selenium (Se), Schneider-Matyka et al. observed a positive correlation between the serum selenium (Se) concentration, HDL cholesterol levels, and age of the studied individuals [138]. Further, while published studies indicate that mineral supplementation (such as selenium (Se) or zinc (Zn)) can significantly alter systemic levels of these elements in older adults, this does not necessarily imply a requirement for routine supplementation in all elderly individuals, especially in the absence of clinical deficiency. For example, long-term zinc (Zn) supplementation in the Age-Related Eye Disease Study significantly altered serum zinc (Zn) levels (AREDS Report No. 7), and selenium (Se) status has been shown to respond to dietary supplementation in older cohorts [139,140]. Nevertheless, the specific impact of routine supplementation on vitreous humor elemental concentrations remains uncharacterized and warrants further study.

#### 3.4.2. BMI

BMI was significantly associated with only a limited subset of elemental measures, namely, negatively correlated with beryllium (Be) and chromium (Cr) concentrations, and one positive correlation, which was caesium (Cs). Particular attention should be drawn to the trend in chromium (Cr) levels (lower concentrations) with increasing obesity. Chromium (Cr) is responsible for the maintenance of glucose, lipid, and insulin metabolism, and it was also reported to increase lean body mass at the same time decreasing percentage body fat, which ultimately might lead to weight loss in overweight individuals [141,142]. It was also noted that a low chromium (Cr) diet significantly affects metabolic parameters, increasing body fat, energy intake, as well as circulating triglycerides and insulin [143]. Thus, inverse association of chromium (Cr) with BMI may reflect metabolic dysregulation commonly observed in overweight and obese individuals [144,145]. Current research indicates that serum levels of chromium (Cr) tend to be lowered in such metabolic diseases as obesity and diabetes [146]. In addition, it was shown that supplementation with chromium ions (III) decreases blood glucose levels, as well as lowers cholesterol and low-density lipoprotein (LPL) levels [146]. It was also indicated that chromium (Cr) and cadmium (Cd) exposure might be associated with BMI and waist circumference [147]. In addition, Uche et al. showed that beryllium (Be) and platinum (Pt) exposure was significantly and positively associated with childhood obesity [148]. Regarding caesium (Cs), the researchers have so far presented another relationship other than the one presented in our study. In the studies devoted to elemental analysis and BMI, the researchers indicated that increased caesium (Cs) levels are rather associated with a decrease in BMI [149].

#### 3.4.3. Alcohol Consumption

The observed elevated zinc (Zn) levels in the vitreous humor of individuals who abused alcohol is an interesting phenomenon which remains contradictory to the current research that indicates that alcoholism leads to low zinc (Zn) levels primarily because of inadequate dietary intake, impaired absorption of zinc (Zn) from food, or increased excretion through urine [150,151]. This observation might indicate that zinc (Zn) presents a preferential accumulation site in the vitreous humor of the human eye. This might be explained by the fact that zinc (Zn) has a fundamental and complex role in the physiology of the human organ of vision, being involved in such processes as the maintenance of cellular homeostasis, visual processing, or antioxidant defense [152,153,154,155]. High concentrations of zinc (Zn) were reported in the retinal pigment epithelium/choroid complex (RPE/choroid), and both excess and deficiency of this element might lead to cellular dysfunction within the organ of vision [152]. The absence of statistically significant differences among females likely reflects the very small number of alcohol-dependent women in the cohort, highlighting a limitation of the study while simultaneously underscoring the importance of sex-stratified analyses in biomedical research.

#### 3.4.4. Sex

Sex-based comparisons revealed statistically significant differences with regard to calcium (Ca) levels; higher concentrations of calcium (Ca) were observed in female subjects compared to males. Sex-related differences obtained in our study remain partially consistent with data found in scientific literature; even though the vitreous humor was not yet compared between females and males in terms of the abovementioned elements, other human samples were. Calcium (Ca) was reported to be higher in females compared to males, especially after menopause, which might be associated with hyperactive parathyroid glands in postmenopausal women [156,157].

## 4. Materials and Methods

### 4.1. Studied Population

**Figure 10:**
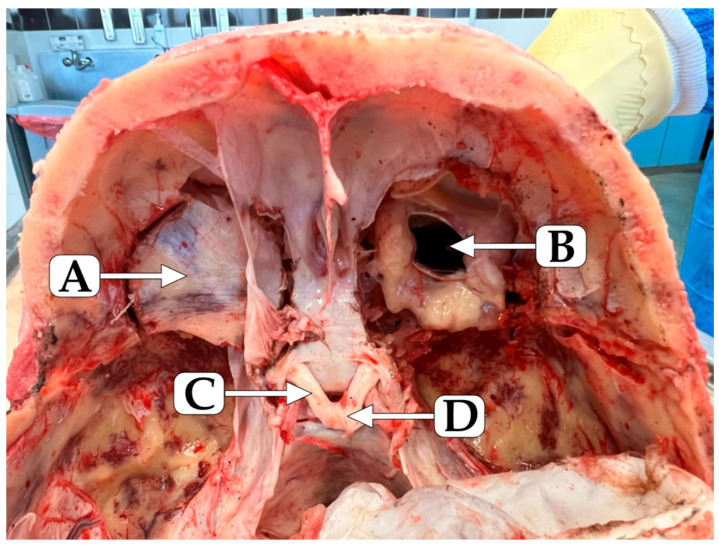
Transverse section of the human head at the level of the optic chiasm and opening of the orbits through the superior walls (the right eyeball cut in coronal section). A—superior wall (cut) of the left orbit; B—opened right eyeball; C—left optic nerve; D—optic chiasm. The picture was done during an autopsy at the Department of Forensic Medicine, Medical University of Lublin, Poland.

The vitreous humor obtained for the metallomic analysis was collected in the Department of Forensic Medicine, Medical University of Lublin. The study was approved by the Local Ethical Committee (Medical University of Lublin, Poland, approval no KE-0254/217/2021) and the sample collection was approved by the Prosecutor’s Office responsible for the performed autopsies. The study was conducted in accordance with the Code of Ethics of the World Medical Association, Declaration of Helsinki, for experiments involving humans. Deceased presenting any signs of decay, as well as those who died in accidents that involved facial injuries, preventing us from collecting the vitreous humor samples, were excluded from the study. Samples were collected from the subjects within a 24-h time window following confirmation of death. The study includes 57 subjects (Supplementary S5) (Figure 10).

Causes of death were grouped into suicide, sudden death, and accident. The cause of death of the individuals was either unintentional (accident, sudden death) or intentional (suicide). The distinction between sudden death and accidental death was based on the circumstances of death. Accidental deaths included events such as traffic accidents and other unintended incidents involving external factors, whereas sudden death referred to unexpected natural deaths not classified as intentional (suicide) or accidental. Deaths due to accidents included deaths from fire, accidents at work/while doing housework, traffic accidents, unintentional falls from height, and drowning. According to the medical information obtained during the autopsies, the most common medical conditions of the deceased included atherosclerosis (primarily of the coronary arteries and aorta in the majority of cases), hepatic steatosis or cirrhosis, gastritis or gastric hemorrhages, and pancreatitis. Information on chronic alcohol abuse was obtained from autopsy reports and available medical history and included as a variable due to its known impact on elemental metabolism. All the information regarding the available medical conditions of the deceased is presented in the Supplementary Materials (Supplementary S1). The demographic characteristics of the studied population are presented in Table 1 and Table 2 (Table 7 and Table 8).

### 4.2. Sample Preparation

Wet mineralization of each sample of a mean mass of 1.5 g (0.12–4.32 g) was conducted using 7 mL of 69% suprapur nitric acid HNO_3_ (Baker, Radnor, PA, USA). Afterwards, heating to 190 °C in closed Teflon containers, the microwave mineralization system TOPEX (PreeKem, Shanghai, China) was performed. After mineralization, in order to stabilize some elements (As, Hg, Se, Mo, Tl, Ag), 1 mL of HCl (Merck, Darmstadt, Germany) was added. Finally, the samples were diluted to 25 mL by ultrapure water obtained in the purification system Milli-Q (Millipore, Darmstadt, Germany). The working standards and blanks were analyzed before each sequence of samples to prepare calibration curves and verify the purity of the chemicals and containers used.

### 4.3. ICP-MS Measurements

The inductively coupled plasma mass spectrometer Agilent 8900 ICP-MS Triple Quad (Agilent, Santa Clara, CA, USA) was used for the elemental analysis of the vitreous humor. Most elements were analyzed in He mode (5.5 mL/min helium flow); selenium (Se) and arsenic (As) were analyzed in O_2_ mode (gas O_2_ flow rate-30%). The plasma was working in general-purpose mode with 1.550 kW RF power, the nebulizer gas flow was 1.07 L/min, the auxiliary gas flow was 0.9 L/min, and the plasma gas flow was 15 L/min. Depending on the predicted concentration of the element, the acquisition time was from 0.1 to 2 s. Because of the lack of certified reference material, the internal standard ISTD (Sc, Y, Lu) with a concentration of 0.5 ppm was used for the analysis. ISTD was added automatically using a standard mixing connector, the so-called mixing tee. The obtained recoveries were in the range of 80–120%. ICP commercial analytical standards were purchased from Agilent Technologies, Santa Clara, CA, USA (Multi-Element Calibration Standard 2A-Hg, Environmental Calibration Standard, Multi-Element Calibration Standard 2A), Merck Millipore, Darmstadt, Germany (ICP- Multi-Element Calibration Standard XVII, ICP- Multi-Element Calibration Standard VI, Phosphorus ICP standard), Honeywell Fluka™ analytical standards (Platinum Standard for ICP, Palladium Standard for ICP), and Inorganic Ventures, Christiansburg, Virginia, US (Rare Earth, Standards). The validation protocol of this analytical method was presented as Supplementary Materials (Supplementary S6). Supplementary S7 presents instrumental and methodological detection and quantitation limits for analyzed elements (Supplementary S7).

### 4.4. Statistics

In the statistical analyses performed in this study, we used different measures of position and dispersion in order to describe the probable distribution of all elements. The statistical analyses were performed using a dataset comprising elemental concentrations quantified in human vitreous humor samples, reported in ppb or ppm depending on the element, together with selected analytical variability indices (RSD, where applicable) and basic anthropometric/clinical variables. Mean and median values were used to describe the central tendency. The dispersion was assessed using minimum and maximum values, standard deviation, and quantiles (25% and 75%). The Spearman rank-order correlation coefficient was used to describe the relationship between the particular elements. The choice of this coefficient was dictated by the strong asymmetry of the distribution of individual elements, and also the fact that the relationships were not always linear. Significances of correlation coefficients were determined by the *t*-test. Cluster analysis was performed to identify homogeneous groups of patients by concentration of elements in the body. This analysis was divided into two stages. The first stage used hierarchical cluster analysis via Ward’s method to estimate the optimal number of clusters. Then, the k-means method was used with the number of clusters adopted from the hierarchical method. The significance of differences between the obtained clusters was determined by the Kruskal–Wallis rank-sum test. A nonparametric test was used because of the abnormal distribution of the analyzed variables. Because elemental concentrations showed pronounced skewness and occasional extreme values, non-parametric procedures were used for inferential analyses, whereas means ± standard deviations are reported for completeness and comparability with previous metallomic studies.

## 5. Limitations and Future Directions

In the following study, several limitations should be considered. Our study was limited to a single biological matrix, as elemental concentrations were assessed only in the vitreous humor. The absence of parallel measurements in blood, urine, or other ocular tissues restricts direct evaluation of inter-compartmental gradients and limits conclusions regarding systemic versus local accumulation processes. Moreover, detailed information on lifetime environmental exposure, occupational history, dietary habits, medication use, and micronutrient supplementation was not available in all of the cases or was highly limited. Further, we acknowledge that alternative measures of adiposity, such as waist circumference, waist-to-hip ratio (WHR), or body fat percentage, may also provide an evaluation of obesity than BMI alone. However, within the forensic setting, the application of these alternative definitions is not feasible, as only basic anthropometric parameters—body length and body weight—are routinely recorded during postmortem examination. Consequently, BMI was used as the sole available indicator of body composition in our study. Although causes of death were categorized, comprehensive data on chronic comorbidities was limited to the data and observations obtained during autopsies since clinical documentation of individual patients was unavailable. Similarly, information regarding pre-existing ocular diseases or ophthalmic conditions was not reported in available medical and autopsy reports. Therefore, stratification according to specific ophthalmic conditions was not feasible. This limitation highlights the need for further studies designed to specifically investigate elemental profiles in vitreous humor in well-characterized cohorts of patients with defined ophthalmic diseases, which may provide further insights into disease-specific alterations in elemental homeostasis. Further, due to the specific characteristics of the examined material (vitreous humor samples collected postmortem from human subjects), the application of standard clinical ophthalmic diagnostic methods investigating axial length or refraction was not feasible. Moreover, instrumental assessment of these parameters could not be performed under forensic mortuary conditions. As the study population originated from a single forensic center in Poland, the cohort can be assumed to represent a relatively ethnically homogenous population of Polish individuals residing in south-eastern Poland (the Lublin region). This may limit the generalizability of the results to other populations, highlighting the need for future studies including ethnically diverse cohorts.

## 6. Conclusions

The present study demonstrates that the human vitreous humor represents a biochemical environment characterized by consistently high concentrations of macroelements, particularly calcium (Ca), sodium (Na), potassium (K), and magnesium (Mg). The observed increasing age-related accumulation of cadmium (Cd) likely reflects chronic environmental exposure and limited elimination. In parallel, elevated levels of copper (Cu) and selenium (Se), increasing with age, and higher zinc (Zn) concentrations observed in individuals with a history of alcoholism may represent adaptive responses linked to antioxidant defense mechanisms. These findings warrant a need for further investigation into the accumulation of toxic, trace, and ultra-trace elements in the pathophysiology of the organ of vision. It should be emphasized that longitudinal and comparative studies are needed to confirm reference ranges for macro- and microelements in vitreous humor and to further explore their role in the function of the eye. Future integrative studies should combine elemental analysis with clinical, molecular, and imaging data.

## Figures and Tables

**Table 1:** Eigenvalues and values explained by the principal components.

Component	Eigenvalue λ	% Variance Explained	Cumulative Variance %
1	12.64	23.88	23.88
2	7.85	14.83	38.71
3	4.72	8.91	47.62
4	3.81	7.21	54.82
5	2.82	5.33	60.16
6	2.79	5.27	65.43
7	2.36	4.46	69.89
8	1.89	3.56	73.45
9	1.60	3.03	76.48
10	1.50	2.83	79.31
11	1.42	2.68	81.99
12	1.19	2.25	84.24
13	1.12	2.11	86.34
14	0.94	1.77	88.11

**Table 2:** Percentage of variance explained and strongest positive and negative loadings for the first thirteen principal components.

Principal Component	% of Variance	Strongest Loadings	
		Positive	Negative
PC1	23.9	Co, Th, Ho, Eu, Er, Sn, Nd, Be, Dy, Sb	-
PC2	14.8	Mn, Al, Sm, Ga	K, P, Ca, Mg, Rb, Cs
PC3	8.9	Ga, Al, Mn, Cu, P, Rb	Na, La, Ce
PC4	7.2	Sb, Pb, V, U, Fe	Zr, Tb, Yb, Nd
PC5	5.3	Hg202, Hg201, Bi, Mo, Se	Tm, Yb, Ti, U, Tl
PC6	5.3	Hg201, Hg202, Bi, Ba, As, Tb, Ti, Sr	Pt, Se
PC7	4.5	Yb, Dy, Th, Mo, Tb, Tl	Cr, Er, Ti, Be
PC8	3.6	Ba, Ti, Tm, Gd, As, Ag, Ca	Hf, Tb, Pd
PC9	3.0	Hg202, Hg201, U, Ti, Fe	Bi, Ag, Pb, Hf, As
PC10	2.8	Ni, Tm, Ho, Pb, Hg201, Hg202	U, Ce, Sr, Fe
PC11	2.7	Ni, Ce, Sr, Gd, Tm	Tl, Ag, Mg, Hf, Eu
PC12	2.2	Tm, Mo, Zn, Cu, Hf	Mg, Sr, Gd, Hg201, Hg202
PC13	2.1	Sr, Tb, Fe, Ag	Hf, Ce, Gd, La, Rb, Tl

**Table 3:** Statistically significant correlations (*p* < 0.05) between elemental concentrations and the age of the studied subjects.

Elements	r	*p*
Ca [ppm]	0.314	0.033
V [ppb]	−0.291	0.049
Cu [ppb]	0.372	0.011
Se [ppb]	0.340	0.021
Cd [ppb]	0.343	0.020
Sb [ppb]	0.355	0.015

**Table 4:** Significant correlations between elemental measures and BMI (*p* < 0.05).

Elements	r	*p*
Be [ppb]	−0.274	0.049
Cr [ppb]	−0.272	0.042
Cs [ppb]	0.282	0.035

**Table 5:** Results of the analysis of sex-related differences in elemental measures in vitreous humor.

Elements	Gender	N	Median	Min	Max	Q1	Q3	Mean	SD	*p*
Ca [ppm]	F	17	64.897	7.1042	108.178	45.9011	76.295	60.934	25.675	*p* = 0.037
Ca [ppm]	M	40	53.7804	9.38655	77.678	38.6294	60.2358	49.1412	17.3101	

**Table 6:** Alcohol-related differences in elemental measures in male subjects.

Elements	Alcoholism (Only Males)	N	Median	Min	Max	Q1	Q3	Mean	SD	*p*
Zn [ppb]	NO	34	255.3824	0.00000	2634.446	0.00000	714.6583	466.4346	593.5012	*p* = 0.02
Zn [ppb]	YES	6	886.4031	417.0149	1498.808	492.0920	1251.013	905.2890	454.839	

**Table 7:** The demographic characteristics of the individuals included in the study.

Demographic characteristics						
Population	Gender	N	%	Min–Max Age	Median Age	Mean Age ± SD
N = 57	Female	17	29.82	17.00–83.00	39.50	48.78 ± 24.20
	Male	40	70.18	20.00–86.00	56.00	56.31 ± 18.23
Demographic characteristics x alcoholism						
Gender		Alcoholism	No alcoholism	Fisher test		
Female		3 (17.65%)	14 (82.35%)	*p* = 0.54245		
Male		6 (15.00%)	34 (85.00%)			
Anthropometric characteristics						
Variable	N	Min–Max	Median	Mean ± SD		
Height (cm)	56	155.0–200.0	173.0	173.44 ± 9.06		
Weight (kg)	56	37.0–115.0	73.0	74.80 ± 16.79		
BMI (kg/m^2^)	56	13.75–36.28	24.23	24.74 ± 4.78		
Cause of death						
Cause of death		N	%			
Suicide		14	24.56			
Sudden death		32	56.14			
Accident		11	19.29			

**Table 8:** BMI categories of the studied population.

BMI Categories *				
BMI	N	Min–Max	Median	Mean ± SD
	56	13.75–36.28	24.23	24.74 ± 4.78
BMI category	N	%		
Underweight	5	8.92		
Normal weight	26	46.42		
Overweight	16	28.57		
Obese class I	7	12.50		
Obese class II	2	3.57		

* BMI categories were defined According to the World Health Organization (WHO) criteria.

## Data Availability

The original contributions presented in this study are included in the article/Supplementary Material. Further inquiries can be directed to the corresponding author(s).

## Supplementary Materials

The following supporting information can be downloaded at: https://www.mdpi.com/article/10.3390/ijms27062527/s1.

## Abbreviations

AAS	atomic absorption spectrometry
AMD	age-related macular degeneration
BMI	body mass index
FAAS	Flame atomic absorption spectrometry
GFAAS	graphite furnace atomic absorption spectrometry
ICP-MS	inductively coupled plasma mass spectrometry
ICP-OES	inductively coupled plasma optical emission spectrometry
PCA	principal component analysis
ppb	parts per billion
ppm	parts per million
WHO	World Health Organization
XRF	X-ray fluorescence
