# Supplementary material for: Metallomic Analysis of Vitreous Humor of the Human Eye—A Post-Mortem Multielemental Study
Source: Int J Mol Sci. 2026 Mar 10;27(6):2527. doi: 10.3390/ijms27062527 (PMC13026291; doi:10.3390/ijms27062527)
Supplement: Supplementary file 1 [file ijms-27-02527-s001.zip › Supplementary 1.pdf]

Element	N	Min	Max	Median	q1	q3	Mean	SD	Skewness	Kurtosis
Ag [ppb]	**57**	0.0000	0.0296	0.0000	0.0000	0.0000	0.0005	0.0039	7.5498	57.0000
Al [ppb]	57	0.0000	42907.0977	1.7951	0.0000	71.3825	907.8153	5687.1650	7.4517	55.9548
As [ppb]	57	0.0000	7.9479	0.0000	0.0000	0.0741	0.2554	1.1708	5.8922	36.1697
Ba [ppb]	57	0.0000	25.0836	0.4155	0.0000	0.9622	1.9414	5.0468	3.7253	13.7371
Be [ppb]	57	0.0000	0.0835	0.0000	0.0000	0.0000	0.0020	0.0115	6.7194	47.0705
Bi [ppb]	57	0.0000	2.2869	0.0000	0.0000	0.0000	0.1360	0.4266	3.7203	14.4527
Ca [ppm]	57	7.1042	108.1776	56.8291	39.0504	65.2869	52.6585	20.6555	-0.1814	0.2000
Cd [ppb]	57	0.0240	40.7639	2.3064	0.5850	7.3620	5.6273	8.5622	2.7941	8.3670
Ce [ppb]	57	0.0000	2.5995	0.0412	0.0000	0.4815	0.3313	0.5274	2.3343	6.2718
Co [ppb]	57	0.0000	1.0430	0.0212	0.0000	0.0755	0.0678	0.1608	4.8793	26.5575
Cr [ppb]	57	0.0000	46.1403	0.0000	0.0000	0.7548	3.0863	9.1573	3.9662	16.4267
Cs [ppb]	57	0.0000	7.4901	2.3846	1.2754	3.4670	2.6493	1.7353	0.8516	0.2249
Cu [ppb]	57	0.0000	285.2108	26.9815	13.8430	47.4214	37.6494	42.2904	3.8490	20.7107
Dy [ppb]	57	0.0000	0.0658	0.0010	0.0000	0.0062	0.0066	0.0136	2.9493	8.8001
Er [ppb]	57	0.0000	0.0969	0.0018	0.0000	0.0092	0.0085	0.0164	3.5533	15.5412
Eu [ppb]	57	0.0000	0.0463	0.0004	0.0000	0.0042	0.0040	0.0083	3.3410	12.8988
Fe [ppb]	57	7.0931	1941.6938	182.9953	89.9397	399.2470	320.2954	379.2349	2.5559	7.6954
Ga [ppb]	57	0.0000	7.1473	0.0000	0.0000	0.0273	0.2152	0.9879	6.5523	45.3988
Gd [ppb]	57	0.0000	0.0469	0.0000	0.0000	0.0021	0.0047	0.0105	2.7905	8.0480
Hf [ppb]	57	0.0000	0.0290	0.0000	0.0000	0.0000	0.0029	0.0069	2.5405	5.7440
Hg 201 [ppb]	57	0.0000	3.2990	0.0392	0.0000	0.1578	0.2306	0.5550	3.9306	17.7102
Hg 202 [ppb]	57	0.0000	3.4084	0.0157	0.0000	0.1100	0.2168	0.5627	4.1487	19.7465
Ho [ppb]	57	0.0000	0.0261	0.0006	0.0000	0.0031	0.0027	0.0049	3.2478	12.0918
K [ppm]	57	105.4411	965.9489	566.1562	456.4188	669.4579	569.1637	171.5943	-0.1231	0.1056
La [ppb]	57	0.0000	0.5613	0.0180	0.0000	0.0872	0.0735	0.1299	2.4176	5.6469
Mg [ppm]	57	3.0363	35.3953	21.3102	17.8332	25.3777	21.5074	5.8473	-0.2131	0.8773
Mn [ppb]	57	0.0000	181.5494	1.7409	0.6084	5.4712	7.3572	24.5612	6.6625	47.2406
Mo [ppb]	57	0.0000	13.6335	0.2281	0.0000	3.2667	2.1592	3.2638	1.7953	2.8392
Na [ppm]	57	394.4628	4240.6066	2818.4038	2644.0599	3043.3151	2830.5506	488.2478	-1.5558	11.5378
Nd [ppb]	57	0.0000	0.3146	0.0016	0.0000	0.0162	0.0240	0.0603	4.0805	17.6037
Ni [ppb]	57	0.0000	24.4023	0.0000	0.0000	0.2273	1.2349	3.7422	4.7906	26.9559

P [ppm]	57	15.2741	191.4519	84.8329	48.0195	119.2484	88.3078	49.2908	0.5619	-0.6641
Pb [ppb]	57	0.0000	21.6819	0.0000	0.0000	0.6654	1.4194	4.1022	4.1055	17.6471
Pd [ppb]	57	0.0000	0.0812	0.0115	0.0000	0.0240	0.0172	0.0210	1.2049	0.4813
Pr [ppb]	57	0.0000	0.1205	0.0034	0.0000	0.0153	0.0118	0.0218	3.5694	14.4520
Pt [ppb]	57	0.0000	0.0663	0.0024	0.0000	0.0083	0.0075	0.0126	2.6388	8.3309
Rb [ppb]	57	147.8198	1482.5616	787.4729	523.7178	966.3735	782.2083	327.2737	0.2865	-0.5083
Sb [ppb]	57	0.0000	1.6528	0.0654	0.0178	0.1924	0.1624	0.2740	3.5551	15.7355
Se [ppb]	57	0.0000	20.9430	5.0790	3.5953	8.3311	6.5122	4.5052	1.2534	1.6222
Sm [ppb]	57	0.0000	0.2226	0.0095	0.0003	0.0169	0.0178	0.0338	4.3858	24.3445
Sn [ppb]	57	0.0000	2.0352	0.0000	0.0000	0.1280	0.1269	0.3284	4.2750	21.3951
Sr [ppb]	57	1.4717	321.0069	37.1582	26.6968	56.2468	52.6064	49.0760	3.2884	15.3314
Tb [ppb]	57	0.0000	0.0217	0.0000	0.0000	0.0010	0.0021	0.0043	2.5971	7.4748
Th [ppb]	57	0.0000	0.0900	0.0000	0.0000	0.0000	0.0046	0.0175	4.2583	17.6954
Ti [ppb]	57	0.0000	224.8881	1.2226	0.2498	5.0340	8.0391	30.2924	6.8321	49.1098
Tl [ppb]	57	0.0000	0.0745	0.0000	0.0000	0.0000	0.0050	0.0142	3.4276	12.4209
Tm [ppb]	57	0.0000	0.0044	0.0000	0.0000	0.0001	0.0004	0.0009	2.8326	8.1499
U [ppb]	57	0.0000	0.1682	0.0000	0.0000	0.0000	0.0067	0.0266	5.2066	28.2072
V [ppb]	57	0.0000	8.0846	0.0634	0.0000	0.3548	0.5519	1.3914	4.0823	18.1691
Yb [ppb]	57	0.0000	0.0348	0.0000	0.0000	0.0000	0.0020	0.0068	3.8975	15.0279
Zn [ppb]	57	0.0000	3636.1493	417.0149	41.2536	929.3256	612.9806	724.9021	1.9563	5.0155
Zr [ppb]	57	0.0000	1.0644	0.0000	0.0000	0.0000	0.0589	0.1828	3.9240	17.3282

Supplementary 1. Descriptive statistics of elemental concentrations in human vitreous humor.
