# Supplementary material for: Metallomic Analysis of Vitreous Humor of the Human Eye—A Post-Mortem Multielemental Study
Source: Int J Mol Sci. 2026 Mar 10;27(6):2527. doi: 10.3390/ijms27062527 (PMC13026291; doi:10.3390/ijms27062527)
Supplement: Supplementary file 1 [file ijms-27-02527-s001.zip › Supplementary 2.pdf]

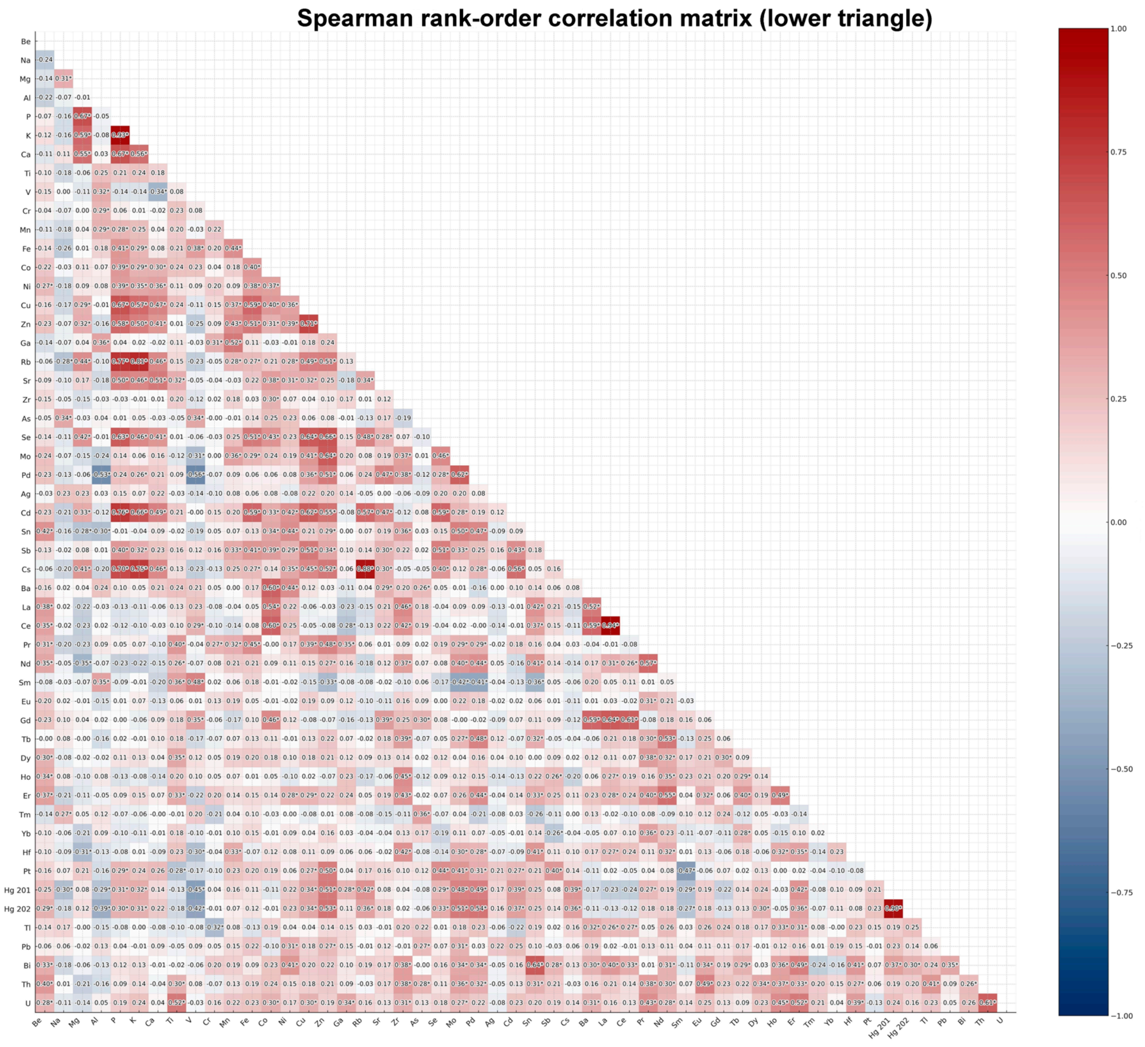

Supplementary 2. Spearman rank-order correlation matrix (lower triangle). Color intensity reflects the strength of the correlations – darker red indicates stronger positive correlations, whereas darker blue indicates stronger negative correlations.
