# Supplementary material for: Metallomic Analysis of Vitreous Humor of the Human Eye—A Post-Mortem Multielemental Study
Source: Int J Mol Sci. 2026 Mar 10;27(6):2527. doi: 10.3390/ijms27062527 (PMC13026291; doi:10.3390/ijms27062527)
Supplement: Supplementary file 1 [file ijms-27-02527-s001.zip › Supplementary 3.pdf]

Elements	PC1	PC2	PC3	PC4	PC5	PC6	PC7	PC8	PC9	PC10	PC11	PC12	PC13
Be [ppb]	0,745	-0,066	-0,226	-0,296	0,216	-0,23	-0,305	-0,087	0,109	0,118	-0,022	-0,151	0,157
Na [ppm]	-0,269	-0,512	-0,509	-0,008	-0,101	-0,275	0,282	0,173	-0,163	0,099	-0,197	-0,094	0,124
Mg [ppm]	-0,142	-0,675	0,07	-0,268	-0,208	-0,113	-0,02	0,225	-0,145	0,063	-0,273	-0,377	0,077
Al [ppb]	0,159	0,736	0,604	-0,072	0,111	-0,007	-0,03	0,023	-0,153	-0,042	0,018	-0,057	-0,028
P [ppm]	0,337	-0,713	0,47	-0,215	-0,128	-0,035	-0,138	0,023	-0,01	-0,067	-0,02	-0,123	0,013
K [ppm]	0,301	-0,777	0,294	-0,114	-0,263	0,04	-0,106	-0,139	0,035	-0,05	-0,061	-0,144	-0,085
Ca [ppm]	0,062	-0,683	0,276	-0,205	-0,024	0,023	0,047	0,257	-0,166	-0,008	0,185	-0,091	-0,182
Ti [ppb]	0,154	-0,096	0,289	-0,185	-0,347	0,374	-0,324	0,454	0,249	-0,051	-0,238	0,174	0,051
V [ppb]	0,546	0,502	0,145	0,45	-0,24	-0,107	-0,036	-0,115	-0,039	0,014	0,094	-0,197	0,151
Cr [ppb]	0,525	0,42	0,323	-0,325	0,28	-0,063	-0,372	0,085	-0,093	0,083	0,028	-0,123	0,072
Mn [ppb]	0,273	0,736	0,557	-0,089	0,022	0,012	0,098	0,041	-0,144	-0,031	-0,043	-0,001	-0,118
Fe [ppb]	0,644	-0,026	0,177	0,379	-0,137	-0,305	0,026	-0,024	0,238	-0,234	0,133	0,148	0,241
Co [ppb]	0,892	-0,072	-0,174	0,218	-0,134	-0,122	-0,052	0,004	0,052	-0,048	-0,08	-0,148	-0,015
Ni [ppb]	0,307	-0,114	0,034	0,113	-0,276	-0,005	-0,256	-0,03	-0,043	0,616	0,504	-0,049	-0,008
Cu [ppb]	0,486	-0,392	0,537	0,11	0,139	-0,186	0,146	0,033	-0,118	-0,047	0,057	0,238	0,016
Zn [ppb]	0,541	-0,533	0,34	-0,041	0,207	-0,111	0,202	-0,005	-0,081	0,024	0,059	0,266	-0,012
Ga [ppb]	0,175	0,69	0,645	-0,104	0,028	0,066	-0,093	0,14	-0,091	-0,027	-0,056	0,003	-0,047
Rb [ppb]	0,178	-0,665	0,417	-0,12	-0,201	0,225	-0,158	-0,167	0,084	0	-0,075	0,042	-0,258
Sr [ppb]	0,292	-0,42	0,07	-0,253	-0,085	0,372	0,078	-0,204	-0,183	-0,209	0,329	-0,287	0,323
Zr [ppb]	0,561	0,012	-0,304	-0,533	0,196	-0,16	-0,057	-0,037	0,083	0,093	-0,019	0,146	-0,153
As [ppb]	0,381	-0,016	-0,191	0,481	0,234	0,415	-0,005	0,322	-0,286	-0,136	0,136	-0,091	0,063
Se [ppb]	0,389	-0,522	0,367	-0,036	0,32	-0,332	0,131	0,138	-0,016	-0,054	0,009	0,074	-0,079
Mo [ppb]	0,626	-0,245	-0,039	0,337	0,346	0,09	0,319	0,132	-0,129	0,113	-0,028	0,266	0,033
Pd [ppb]	0,535	-0,361	-0,041	-0,183	0,277	0,291	-0,008	-0,26	-0,225	-0,144	0,056	0,154	0,152
Ag [ppb]	-0,027	-0,201	0,108	0,041	0,128	-0,28	0,256	0,283	-0,353	0,136	-0,292	-0,183	0,21
Cd [ppb]	0,644	-0,411	0,259	0,338	-0,025	-0,114	0,005	-0,032	0,107	-0,016	0,108	-0,007	0,08
Sn [ppb]	0,785	-0,079	-0,246	-0,087	0,245	-0,106	-0,264	-0,163	-0,025	0,052	0,024	0,016	0,147
Sb [ppb]	0,695	-0,178	0,142	0,539	-0,049	-0,259	0,042	-0,131	0,055	-0,086	0,041	0,008	-0,018

Cs [ppb]	0,135	-0,6	0,415	-0,078	-0,301	0,3	-0,085	-0,177	0,105	0,06	-0,029	0,006	-0,188
Ba [ppb]	0,492	-0,027	-0,035	0,286	-0,131	0,424	-0,197	0,617	0,073	-0,154	-0,076	0,06	0,027
La [ppb]	0,636	0,06	-0,468	-0,161	0,031	-0,073	-0,043	0,01	0,01	-0,141	0,172	-0,008	-0,221
Ce [ppb]	0,546	0,054	-0,455	-0,104	0,012	-0,073	-0,07	0,06	0,061	-0,323	0,33	-0,051	-0,283
Pr [ppb]	0,553	0,579	0,327	-0,336	-0,08	0,029	0,263	0,087	0,008	0,028	-0,043	0,13	-0,009
Nd [ppb]	0,746	0,234	-0,171	-0,454	0,051	-0,052	0,226	0,053	0,095	0,096	-0,061	-0,019	-0,039
Sm [ppb]	0,316	0,723	0,441	0,101	-0,115	0	0,088	-0,102	-0,075	0,045	0,023	-0,197	-0,047
Eu [ppb]	0,813	0,187	-0,144	-0,192	0,065	-0,03	-0,187	-0,007	-0,032	0,106	-0,245	-0,015	0,125
Gd [ppb]	0,45	0,083	-0,39	-0,252	-0,04	0,033	0,266	0,376	-0,013	-0,148	0,315	-0,255	-0,274
Tb [ppb]	0,273	-0,082	-0,101	-0,493	-0,177	0,391	0,314	-0,274	-0,092	-0,064	0,005	0,19	0,279
Dy [ppb]	0,705	0,441	0,283	-0,141	-0,088	0,045	0,326	-0,077	0,009	-0,003	-0,024	-0,199	-0,028
Ho [ppb]	0,82	0,069	-0,323	-0,065	-0,004	-0,12	-0,125	-0,139	-0,002	0,18	-0,213	0,015	0,047
Er [ppb]	0,788	-0,033	-0,122	-0,308	0,028	0,106	-0,341	0,155	0,159	0,068	-0,171	0,007	0,046
Tm [ppb]	0,007	0,088	-0,058	-0,085	-0,382	0,02	0,15	0,397	0,175	0,544	0,315	0,275	0,175
Yb [ppb]	0,162	0,046	-0,044	-0,381	-0,367	0,33	0,571	-0,091	-0,004	-0,072	0,051	0,061	0,132
Hf [ppb]	0,348	0,055	-0,217	0,293	-0,25	0,157	-0,144	-0,344	-0,316	0,122	-0,249	0,22	-0,342
Pt [ppb]	0,09	-0,403	0,286	0,081	0,183	-0,488	0,227	0,108	0,019	0,112	0,056	0,002	-0,128
Hg 201 [ppb]	0,074	-0,145	0,117	0,261	0,537	0,48	0,241	-0,072	0,449	0,214	-0,085	-0,201	-0,049
Hg 202 [ppb]	0,079	-0,126	0,084	0,233	0,558	0,447	0,26	-0,099	0,463	0,211	-0,064	-0,224	-0,027
Tl [ppb]	0,518	0,074	-0,259	0,236	-0,329	0,066	0,391	0,015	-0,044	0,032	-0,296	-0,051	-0,216
Pb [ppb]	0,452	-0,055	-0,065	0,5	-0,282	0,266	-0,086	-0,114	-0,337	0,302	0,021	-0,138	-0,018
Bi [ppb]	0,289	-0,081	-0,2	0,163	0,42	0,465	-0,178	0,165	-0,385	-0,008	-0,016	0,129	-0,035
Th [ppb]	0,839	0,064	-0,243	0,03	-0,214	-0,054	0,321	0,014	0,079	0,076	-0,116	-0,019	-0,084
U [ppb]	0,471	0,055	-0,096	0,443	-0,332	-0,099	-0,053	0,04	0,293	-0,343	-0,09	0,042	0,195

Supplementary 3. Loadings of elemental variables for principal components PC1-PC13.
