# Supplementary material for: Metallomic Analysis of Vitreous Humor of the Human Eye—A Post-Mortem Multielemental Study
Source: Int J Mol Sci. 2026 Mar 10;27(6):2527. doi: 10.3390/ijms27062527 (PMC13026291; doi:10.3390/ijms27062527)
Supplement: Supplementary file 1 [file ijms-27-02527-s001.zip › Supplementary 6.pdf]

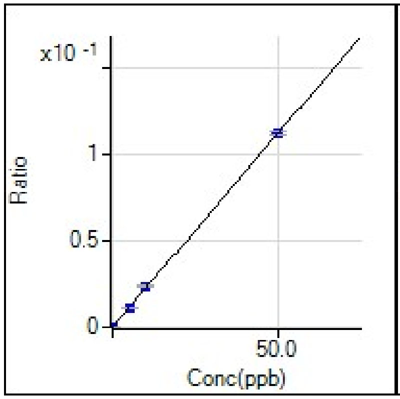

9 Be [No Gas]
ISTD: 45 Sc
y = 2.236E-3 x + 3.452E-5

R	0.9999
DL	0.00551
BEC	0.01544

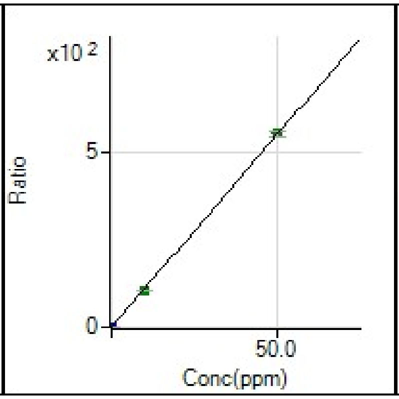

23 Na [He]
ISTD: 45 Sc
y = 1.104E1 x + 1.884E0

R	0.9999
DL	0.004886
BEC	0.1706

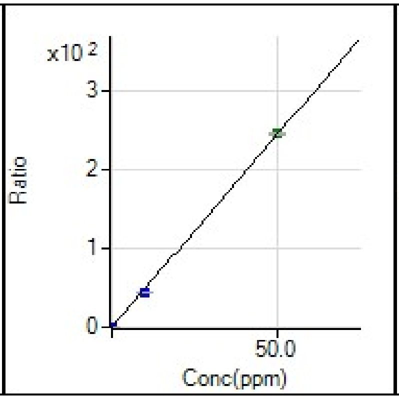

24 Mg [He]
ISTD: 45 Sc
y = 4.899E0 x + 1.451E-2

R	0.9997
DL	0.0007222
BEC	0.002962

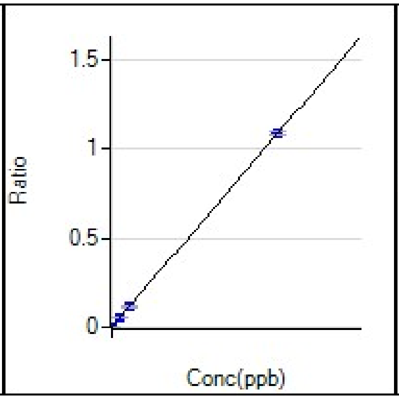

27 Al [He]
ISTD: 45 Sc
y = 1.083E-3 x + 2.265E-3

R	1.0000
DL	0.6479
BEC	2.091

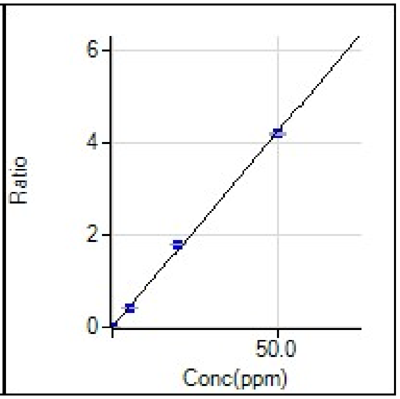

31 P [He]
ISTD: 45 Sc
y = 8.445E-2 x + 8.771E-4

R	0.9995
DL	0.005341
BEC	0.01039

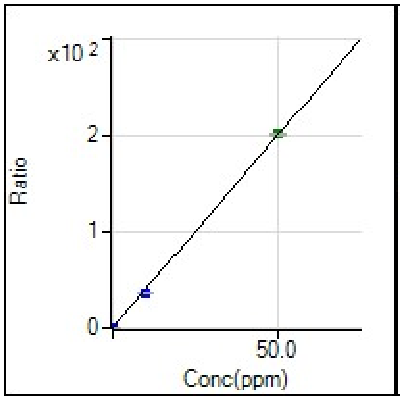

39 K [He]
ISTD: 45 Sc
y = 4.006E0 x + 2.624E-1

R	0.9996
DL	0.002487
BEC	0.06551

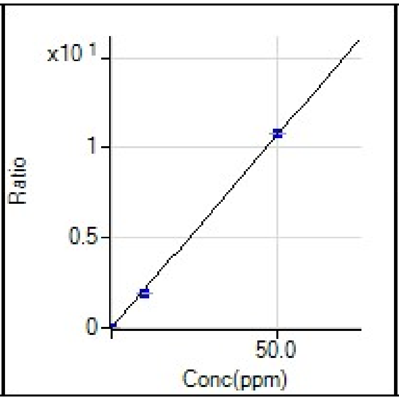

44 Ca [He]
ISTD: 45 Sc
y = 2.140E-1 x + 1.051E-2

R	0.9997
DL	0.01345
BEC	0.0491

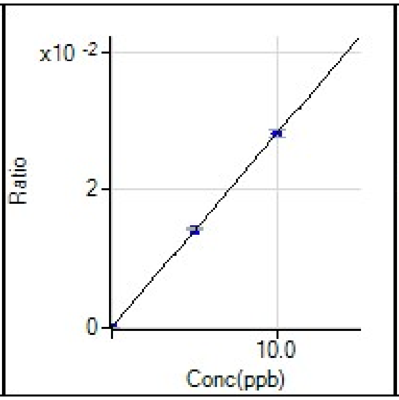

47 Ti [He]
ISTD: 45 Sc
y = 2.808E-3 x + 1.039E-4

R	0.9999
DL	0.04039
BEC	0.03702

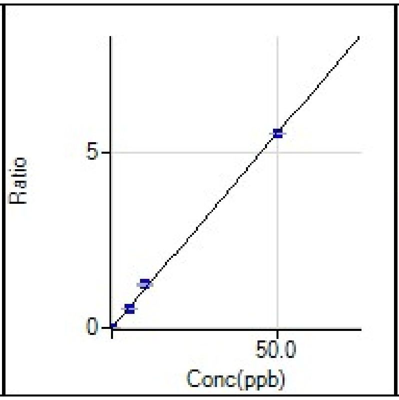

51 V [He]
ISTD: 45 Sc
y = 1.107E-1 x + 9.310E-4

R	0.9996
DL	0.005061
BEC	0.008411

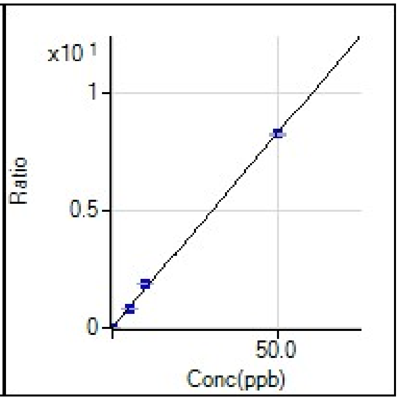

52 Cr [He]
ISTD: 45 Sc
y = 1.653E-1 x + 2.015E-2

R	0.9996
DL	0.02744
BEC	0.1219

Calibration for D:\ICPMH\1\DATA\ciało szkliste_10-04-2025.b\028QBLK.d

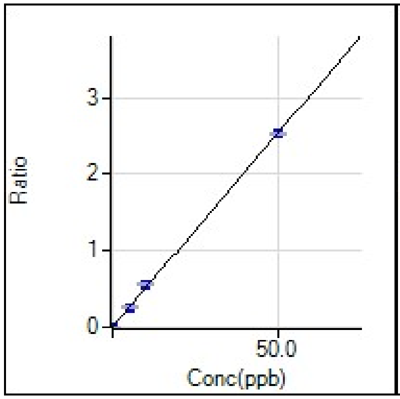

55 Mn [He]
ISTD: 45 Sc
y = 5.077E-2 x + 3.534E-3

R	0.9998
DL	0.0203
BEC	0.06961

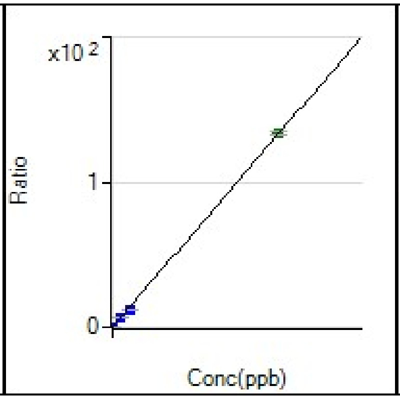

56 Fe [He]
ISTD: 45 Sc
y = 1.330E-1 x + 2.523E-1

R	0.9999
DL	0.06486
BEC	1.897

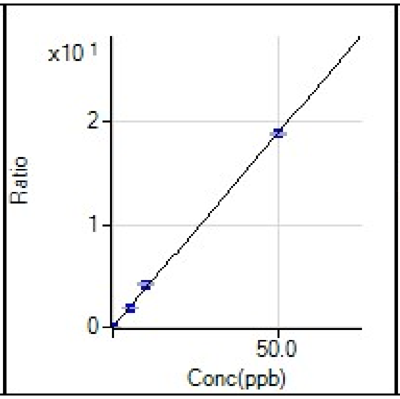

59 Co [He]
ISTD: 45 Sc
y = 3.781E-1 x + 4.870E-3

R	0.9997
DL	0.004856
BEC	0.01288

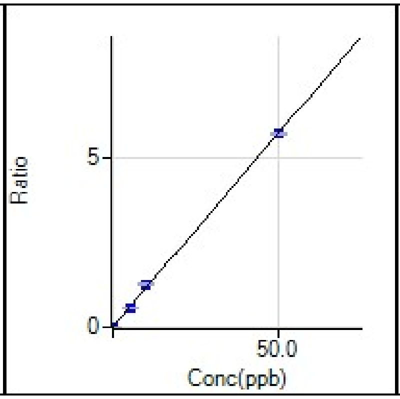

60 Ni [He]
ISTD: 45 Sc
y = 1.140E-1 x + 1.642E-2

R	0.9997
DL	0.04057
BEC	0.1441

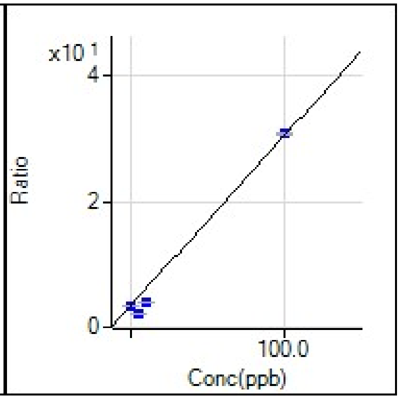

63 Cu [He]
ISTD: 45 Sc
y = 2.707E-1 x + 3.371E0

R	0.9961
DL	0.5695
BEC	12.45

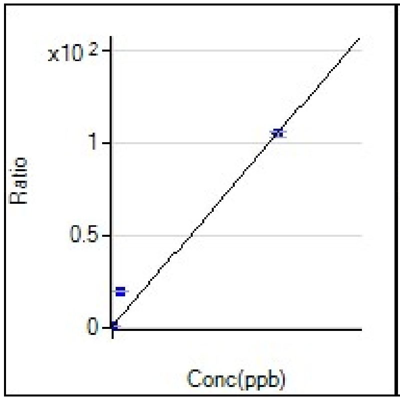

66 Zn [He]
ISTD: 89 Y
y = 1.042E-1 x + 9.982E-1

R	0.9923
DL	1.018
BEC	9.579

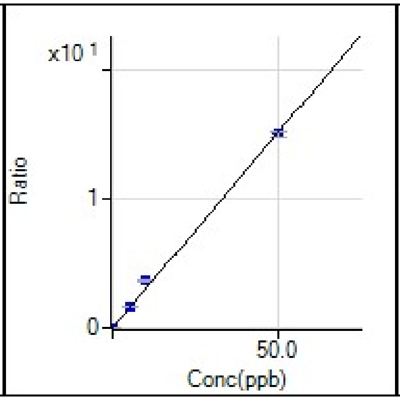

71 Ga [He]
ISTD: 89 Y
y = 3.042E-1 x + 5.718E-3

R	0.9990
DL	0.05141
BEC	0.0188

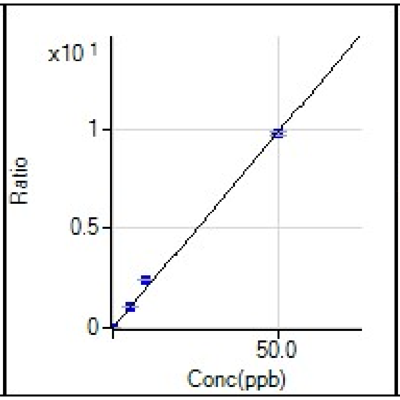

75 As [He]
ISTD: 89 Y
y = 1.972E-1 x + 4.519E-3

R	0.9989
DL	0.02379
BEC	0.02292

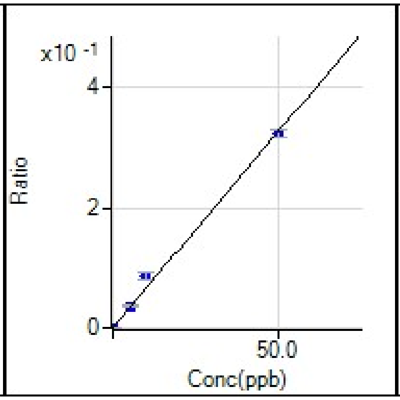

78 Se [He]
ISTD: 89 Y
y = 6.521E-3 x + 8.049E-4

R	0.9978
DL	0.1807
BEC	0.1234

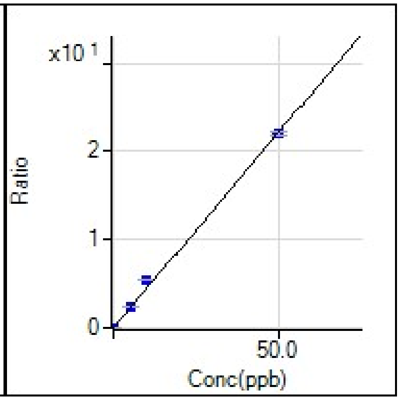

85 Rb [He]
ISTD: 89 Y
y = 4.443E-1 x + 4.648E-3

R	0.9988
DL	0.03582
BEC	0.01046

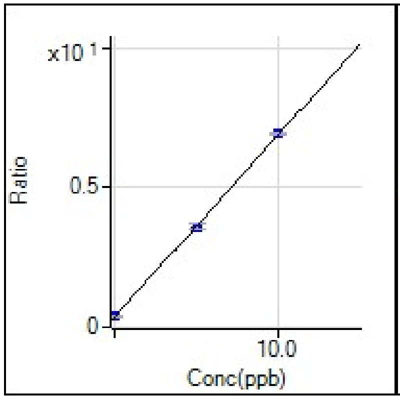

88 Sr [He]
ISTD: 89 Y
y = 6.522E-1 x + 3.797E-1

R	1.0000
DL	0.1154
BEC	0.5822

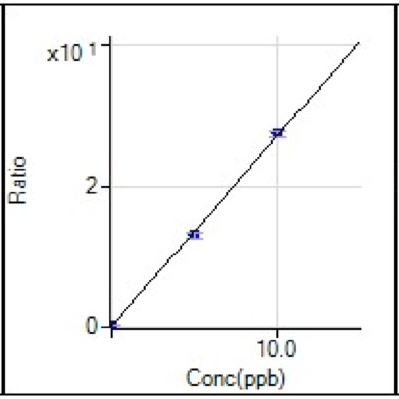

90 Zr [He]
ISTD: 89 Y
y = 2.695E0 x + 1.671E-1

R	0.9994
DL	0.03567
BEC	0.062

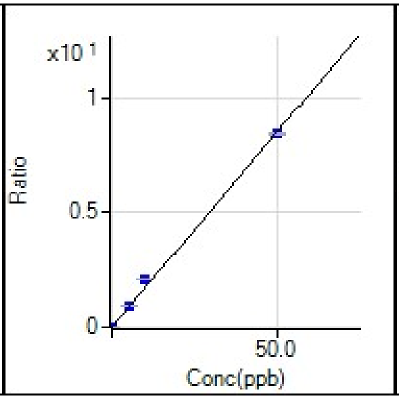

75 -> 91 As [O2]
ISTD: 89 -> 105 Y
y = 1.704E-1 x + 4.153E-3

R	0.9990
DL	0.006188
BEC	0.02437

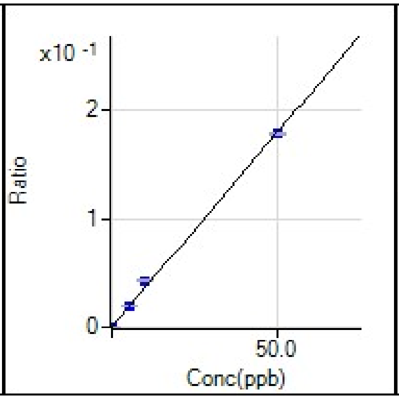

78 -> 94 Se [O2]
ISTD: 89 -> 105 Y
y = 3.589E-3 x + 4.259E-4

R	0.9991
DL	0.08295
BEC	0.1187

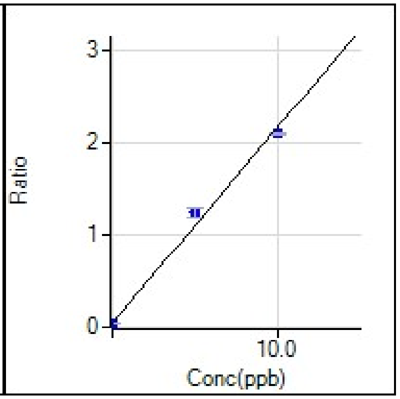

95 Mo [He]
ISTD: 89 Y
y = 2.129E-1 x + 4.306E-2

R	0.9953
DL	0.08348
BEC	0.2023

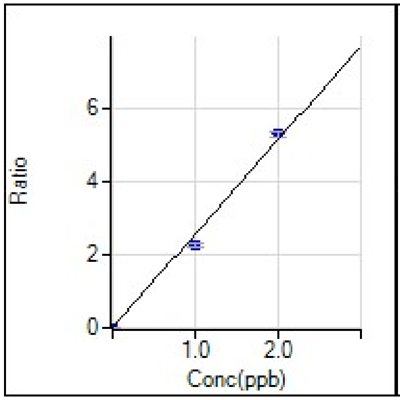

105 Pd [He]
ISTD: 89 Y
y = 2.559E0 x + 1.131E-2

R	0.9962
DL	0.0002653
BEC	0.004421

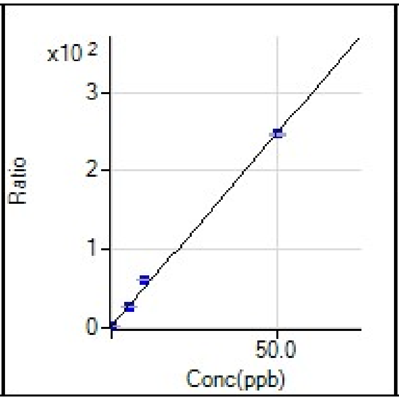

107 Ag [He]
ISTD: 89 Y
y = 4.904E0 x + 3.069E0

R	0.9992
DL	0.06336
BEC	0.6258

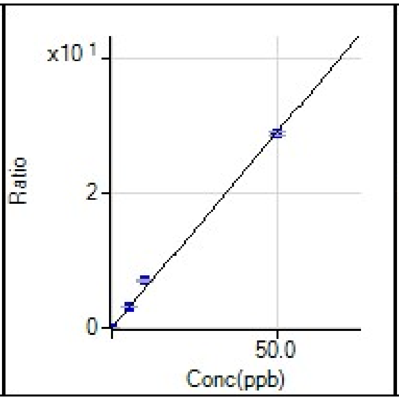

111 Cd [He]
ISTD: 89 Y
y = 5.801E-1 x + 8.551E-3

R	0.9990
DL	0.01464
BEC	0.01474

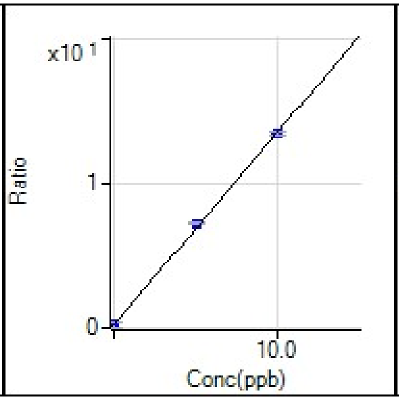

118 Sn [He]
ISTD: 89 Y
y = 1.340E0 x + 2.063E-1

R	0.9993
DL	0.7284
BEC	0.1539

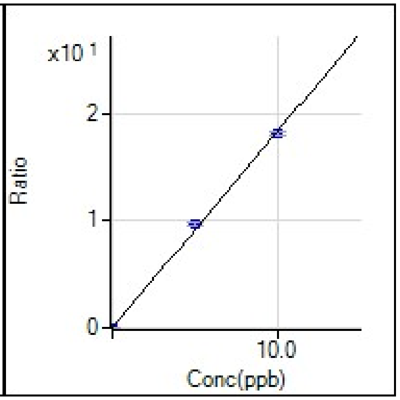

121 Sb [He]
ISTD: 89 Y
y = 1.830E0 x + 2.001E-2

R	0.9992
DL	0.005412
BEC	0.01094

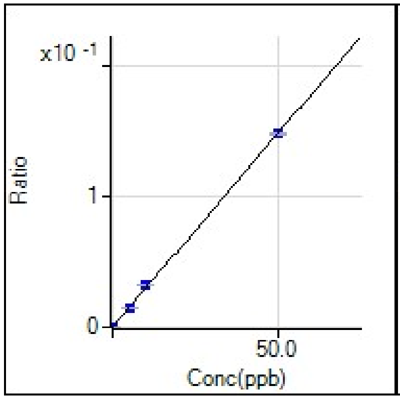

133 Cs [He]
ISTD: 175 Lu
y = 2.958E-3 x + 5.978E-6

R	0.9998
DL	0.002059
BEC	0.002021

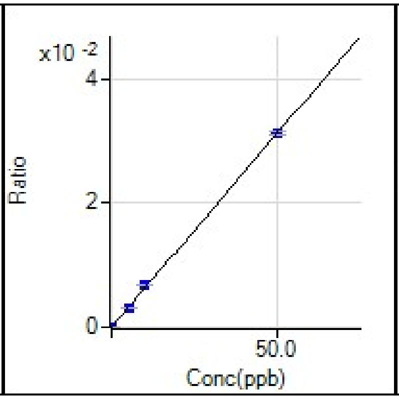

137 Ba [He]
ISTD: 175 Lu
y = 6.247E-4 x + 2.319E-5

R	0.9998
DL	0.0286
BEC	0.03713

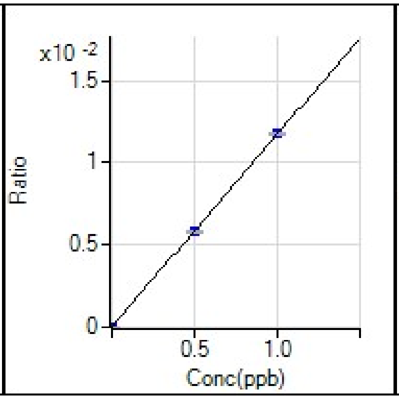

139 La [He]
ISTD: 175 Lu
y = 1.170E-2 x + 2.585E-5

R	1.0000
DL	0.001317
BEC	0.00221

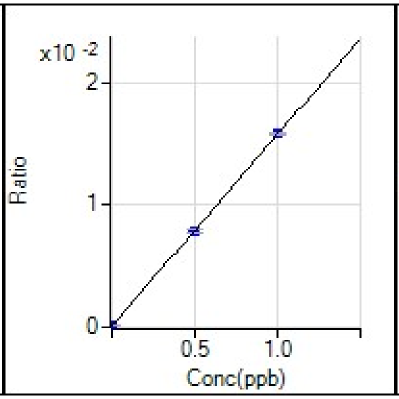

140 Ce [He]
ISTD: 175 Lu
y = 1.566E-2 x + 9.161E-5

R	1.0000
DL	0.002809
BEC	0.00585

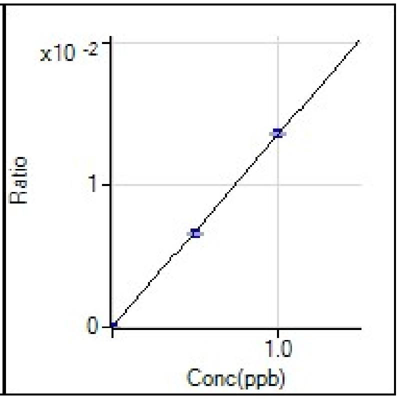

141 Pr [He]
ISTD: 175 Lu
y = 1.345E-2 x + 2.389E-5

R	0.9998
DL	0.001603
BEC	0.001776

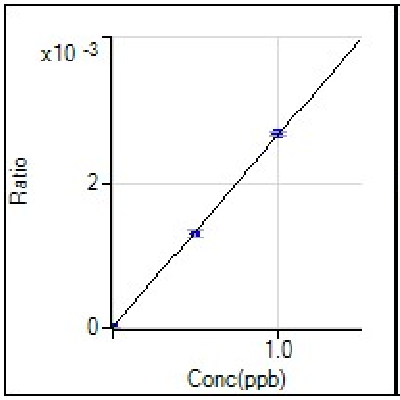

146 Nd [He]
ISTD: 175 Lu
y = 2.649E-3 x + 7.952E-6

R	0.9999
DL	0.005919
BEC	0.003001

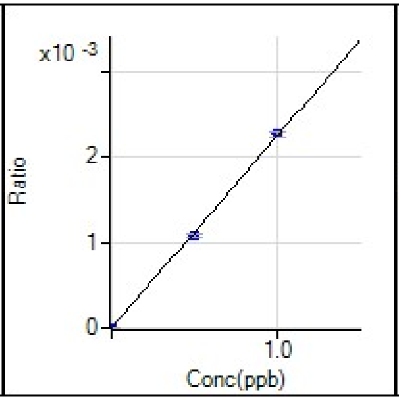

147 Sm [He]
ISTD: 175 Lu
y = 2.250E-3 x + 1.981E-6

R	0.9996
DL	0.004576
BEC	0.0008806

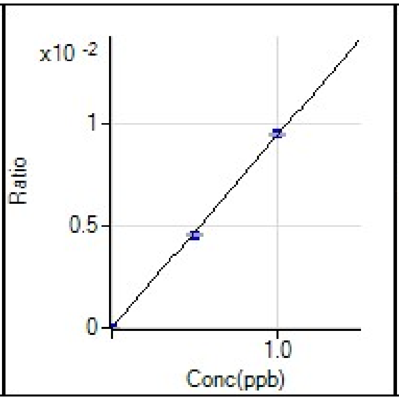

153 Eu [He]
ISTD: 175 Lu
y = 9.394E-3 x + 1.654E-5

R	0.9997
DL	0.002976
BEC	0.00176

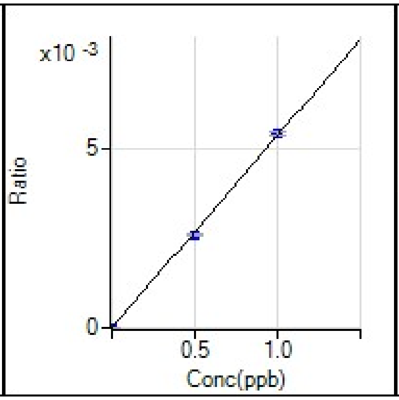

157 Gd [He]
ISTD: 175 Lu
y = 5.343E-3 x + 9.290E-6

R	0.9997
DL	0.001708
BEC	0.001739

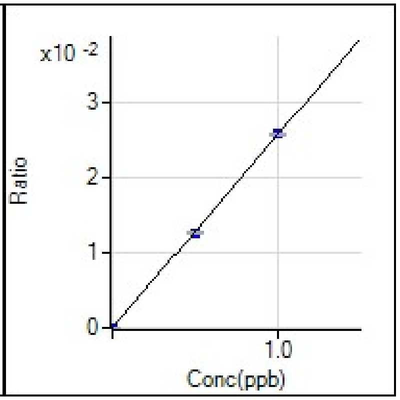

159 Tb [He]
ISTD: 175 Lu
y = 2.573E-2 x + 5.513E-5

R	0.9999
DL	0.002251
BEC	0.002143

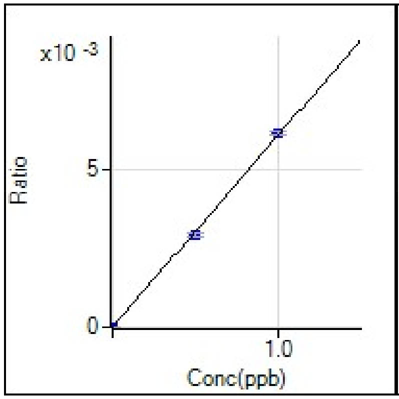

163 Dy [He]
ISTD: 175 Lu
y = 6.047E-3 x + 1.193E-5
R 0.9997
DL 0.002588
BEC 0.001973

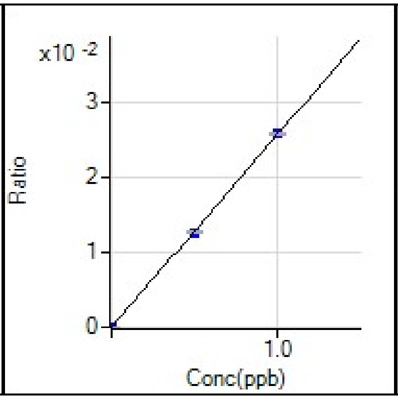

165 Ho [He]
ISTD: 175 Lu
y = 2.573E-2 x + 5.568E-5
R 0.9999
DL 0.0009671
BEC 0.002164

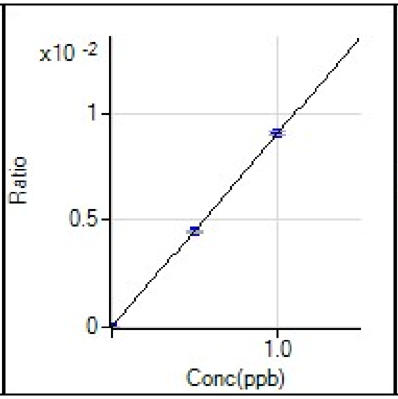

166 Er [He]
ISTD: 175 Lu
y = 8.994E-3 x + 2.254E-5
R 1.0000
DL 0.0009719
BEC 0.002506

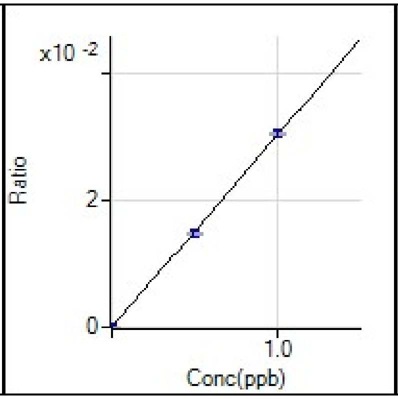

169 Tm [He]
ISTD: 175 Lu
y = 3.035E-2 x + 5.501E-5
R 0.9998
DL 0.0009341
BEC 0.001813

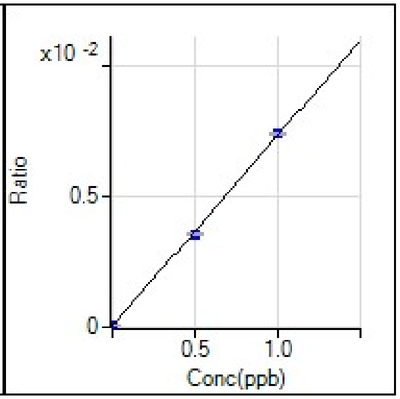

172 Yb [He]
ISTD: 175 Lu
y = 7.265E-3 x + 4.779E-5
R 0.9996
DL 0.003669
BEC 0.006577

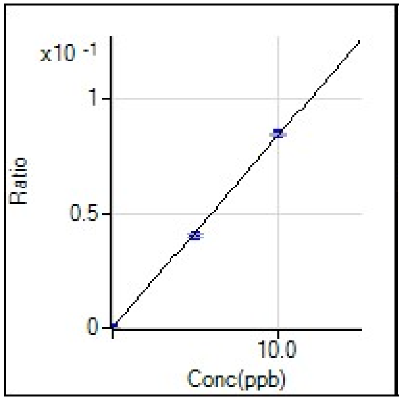

178 Hf [He]
ISTD: 175 Lu
y = 8.387E-3 x + 1.527E-5
R 0.9997
DL 0.001527
BEC 0.001821

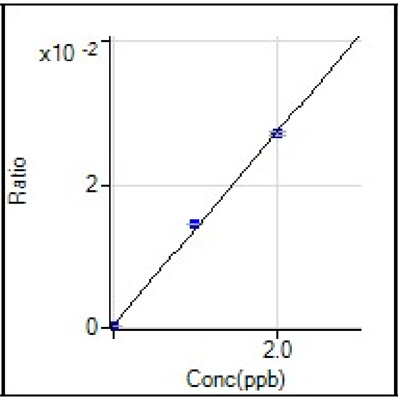

195 Pt [He]
ISTD: 175 Lu
y = 1.355E-2 x + 3.180E-4
R 0.9993
DL 0.006002
BEC 0.02347

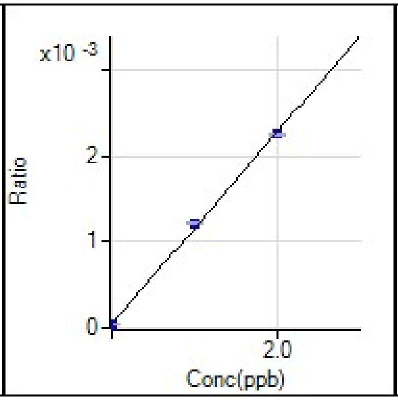

201 Hg [He]
ISTD: 175 Lu
y = 1.115E-3 x + 5.077E-5
R 0.9995
DL 0.01713
BEC 0.04556

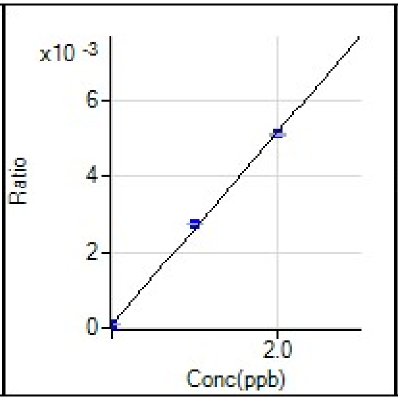

202 Hg [He]
ISTD: 175 Lu
y = 2.535E-3 x + 1.112E-4
R 0.9995
DL 0.001664
BEC 0.04389

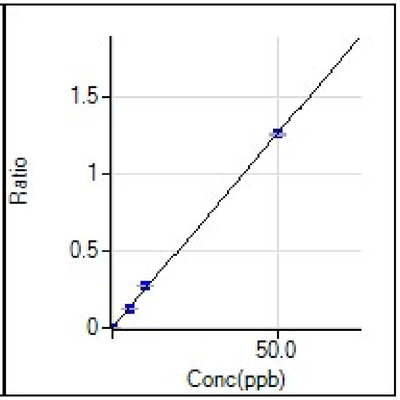

205 Tl [He]
ISTD: 175 Lu
y = 2.528E-2 x + 8.911E-4
R 0.9999
DL 0.001742
BEC 0.03525

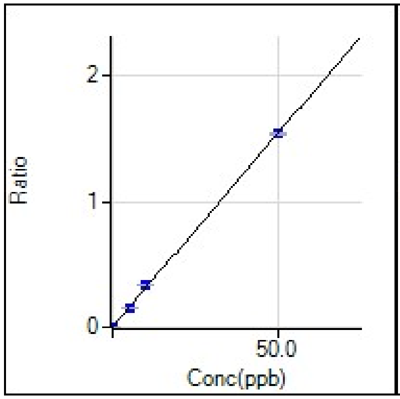

208 Pb [He]
ISTD: 175 Lu
y = 3.085E-2 x + 1.601E-3
R 0.9998
DL 0.01133
BEC 0.0519

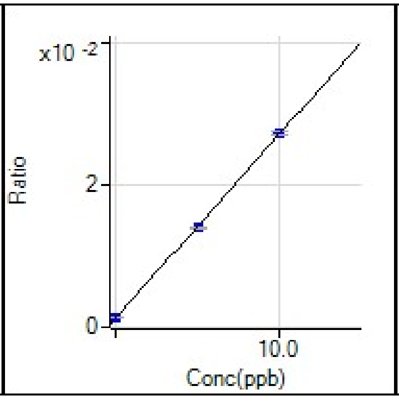

209 Bi [He]
ISTD: 175 Lu
y = 2.563E-3 x + 1.362E-3
R 0.9999
DL 0.09931
BEC 0.5315

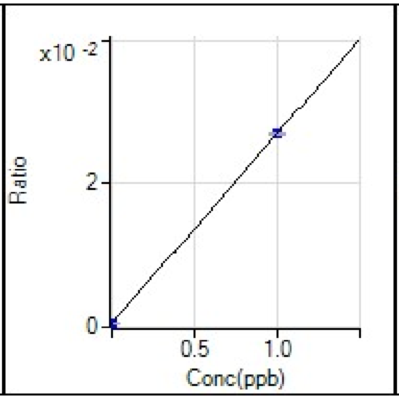

232 Th [He]
ISTD: 175 Lu
y = 2.643E-2 x + 5.573E-4
R 1.0000
DL 0.006735
BEC 0.02109

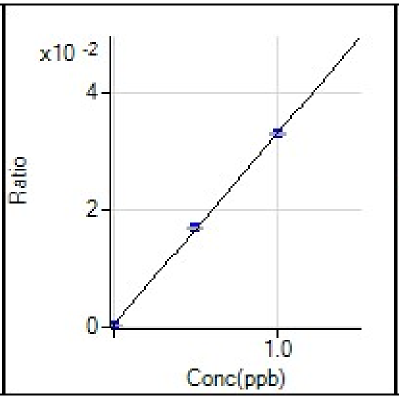

238 U [He]
ISTD: 175 Lu
y = 3.271E-2 x + 3.005E-4
R 1.0000
DL 0.00187
BEC 0.009187

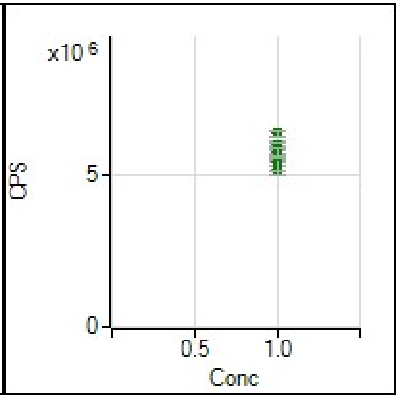

45 Sc [No Gas]
ISTD: ---
Excluded
R
DL
BEC

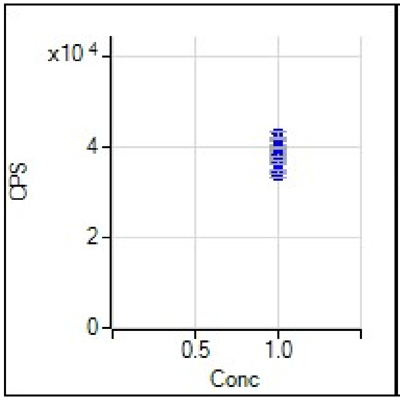

45 Sc [He]
ISTD: ---
Excluded
R
DL
BEC

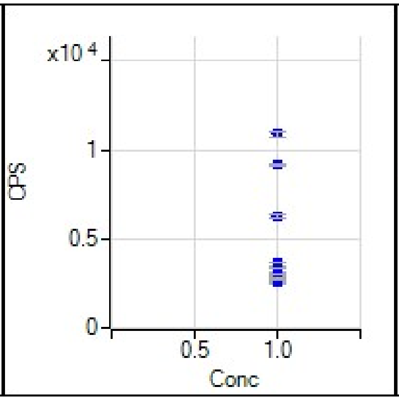

89 Y [He]
ISTD: ---
Excluded
R
DL
BEC

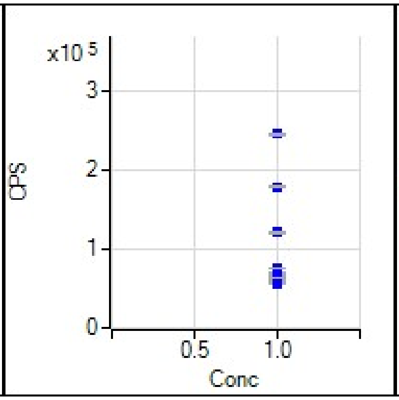

89 -> 105 Y [O2]
ISTD: ---
Excluded
R
DL
BEC

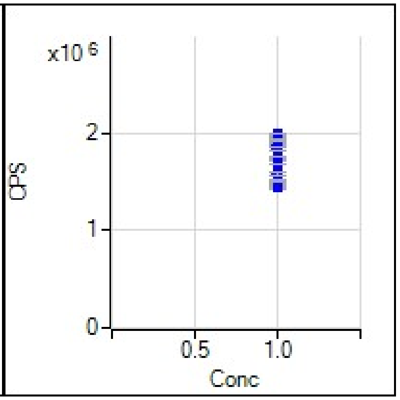

175 Lu [He]
ISTD: ---
Excluded
R
DL
BEC
