# Supplementary material for: Metallomic Analysis of Vitreous Humor of the Human Eye—A Post-Mortem Multielemental Study
Source: Int J Mol Sci. 2026 Mar 10;27(6):2527. doi: 10.3390/ijms27062527 (PMC13026291; doi:10.3390/ijms27062527)
Supplement: Supplementary file 1 [file ijms-27-02527-s001.zip › Supplementary 7.pdf]

Supplementary 7. Instrumental and Methodological Detection and Quantitation Limits for analyzed elements.

Abbreviations and Definitions: IDL (Instrument Detection Limit); LOD (Limit of Detection); LOQ (Limit of Quantitation); min/max/mean: Represent the range and average of detection limits calculated individually for each sample based on its specific mass (m=0.1209-4.3186 g) and constant final volume (V = 25 ml). IDL was determined by the spectrometer manufacturer's protocol as 3 x SD of the calibration blank; LOD was calculated for each individual sample by adjusting the IDL for the sample-specific dilution factor (DF): LOD = IDL x (V/m); LOQ was defined as 3 x LOD.

**Element**	**Unit**	**IDL**	**LOD**			**LOQ**		
			**Mean**	**Min**	**Max**	**Mean**	**Min**	**Max**
107 Ag	[µg/kg]	0.0634	1.6684	0.3668	13.1017	5.0051	1.1004	39.3052
27 Al	[µg/kg]	0.6479	17.0603	3.7506	133.9744	51.1810	11.2519	401.9231
75 -> 91 As	[µg/kg]	0.0062	0.1629	0.0358	1.2796	0.4888	0.1075	3.8387
137 Ba	[µg/kg]	0.0286	0.7531	0.1656	5.9140	2.2593	0.4967	17.7419
9 Be	[µg/kg]	0.0055	0.1451	0.0319	1.1394	0.4353	0.0957	3.4181
209 Bi	[µg/kg]	0.0993	2.6150	0.5749	20.5356	7.8450	1.7247	61.6067
44 Ca	[mg/kg]	0.0135	0.3542	0.0779	2.7812	1.0625	0.2336	8.3437
111 Cd	[µg/kg]	0.0146	0.3855	0.0847	3.0273	1.1565	0.2542	9.0819
140 Ce	[µg/kg]	0.0028	0.0740	0.0163	0.5809	0.2219	0.0488	1.7426
59 Co	[µg/kg]	0.0049	0.1279	0.0281	1.0041	0.3836	0.0843	3.0124
52 Cr	[µg/kg]	0.0274	0.7225	0.1588	5.6741	2.1676	0.4765	17.0223
133 Cs	[µg/kg]	0.0021	0.0542	0.0119	0.4258	0.1627	0.0358	1.2773
63 Cu	[µg/kg]	0.5695	14.9959	3.2968	117.7626	44.9878	9.8904	353.2878
163 Dy	[µg/kg]	0.0026	0.0681	0.0150	0.5352	0.1778	0.0391	1.3964
166 Er	[µg/kg]	0.001	0.0256	0.0056	0.2010	0.0768	0.0169	0.6029
153 Eu	[µg/kg]	0.003	0.0784	0.0172	0.6154	0.2351	0.0517	1.8462
56 Fe	[µg/kg]	0.0649	1.7079	0.3755	13.4119	5.1236	1.1264	40.2357
71 Ga	[µg/kg]	0.0514	1.3537	0.2976	10.6307	4.0611	0.8928	31.8921
157 Gd	[µg/kg]	0.0017	0.0450	0.0099	0.3532	0.1349	0.0297	1.0596
178 Hf	[µg/kg]	0.0015	0.0402	0.0088	0.3158	0.1206	0.0265	0.9473
201 Hg	[µg/kg]	0.0171	0.4511	0.0992	3.5422	1.3532	0.2975	10.6266
202 Hg	[µg/kg]	0.0017	0.0438	0.0096	0.3441	0.1314	0.0289	1.0323
165 Ho	[µg/kg]	0.001	0.0255	0.0056	0.2000	0.0764	0.0168	0.5999
39 K	[mg/kg]	0.0025	0.0655	0.0144	0.5143	0.1965	0.0432	1.5428
139 La	[µg/kg]	0.0013	0.0347	0.0076	0.2723	0.1040	0.0229	0.8170
24 Mg	[mg/kg]	0.0007	0.0190	0.0042	0.1493	0.0571	0.0125	0.4480
55 Mn	[µg/kg]	0.0203	0.5345	0.1175	4.1977	1.6036	0.3525	12.5931
95 Mo	[µg/kg]	0.0835	2.1982	0.4833	17.2622	6.5945	1.4498	51.7866
23 Na	[mg/kg]	0.0049	0.1287	0.0283	1.0103	0.3860	0.0849	3.0310

146 Nd	[µg/kg]	0.0059	0.1559	0.0343	1.2239	0.4676	0.1028	3.6718
60 Ni	[µg/kg]	0.0406	1.0683	0.2349	8.3892	3.2048	0.7046	25.1675
31 P	[mg/kg]	0.0053	0.1406	0.0309	1.1044	0.4219	0.0928	3.3133
208 Pb	[µg/kg]	0.0113	0.2983	0.0656	2.3428	0.8950	0.1968	7.0285
105 Pd	[µg/kg]	0.0003	0.0070	0.0015	0.0549	0.0210	0.0046	0.1646
141 Pr	[µg/kg]	0.0016	0.0422	0.0093	0.3315	0.1266	0.0278	0.9944
195 Pt	[µg/kg]	0.006	0.1580	0.0347	1.2411	0.4741	0.1042	3.7233
85 Rb	[µg/kg]	0.0358	0.9432	0.2074	7.4069	2.8296	0.6221	22.2208
121 Sb	[µg/kg]	0.0054	0.1425	0.0313	1.1191	0.4275	0.0940	3.3573
78 -> 94 Se	[µg/kg]	0.083	2.1842	0.4802	17.1526	6.5527	1.4406	51.4578
147 Sm	[µg/kg]	0.0046	0.1205	0.0265	0.9462	0.3615	0.0795	2.8387
118 Sn	[µg/kg]	0.7284	19.1801	4.2166	150.6203	57.5402	12.6499	451.8610
88 Sr	[µg/kg]	0.1154	3.0387	0.6680	23.8627	9.1161	2.0041	71.5881
159 Tb	[µg/kg]	0.0023	0.0593	0.0130	0.4655	0.1778	0.0391	1.3964
232 Th	[µg/kg]	0.0067	0.1773	0.0390	1.3927	0.5320	0.1170	4.1780
47 Ti	[µg/kg]	0.0404	1.0635	0.2338	8.3519	3.1906	0.7014	25.0558
205 Tl	[µg/kg]	0.0017	0.0459	0.0101	0.3602	0.1376	0.0303	1.0806
169 Tm	[µg/kg]	0.0009	0.0246	0.0054	0.1932	0.0738	0.0162	0.5795
238 U	[µg/kg]	0.0019	0.0492	0.0108	0.3867	0.1477	0.0325	1.1600
51 V	[µg/kg]	0.0051	0.1333	0.0293	1.0465	0.3998	0.0879	3.1396
172 Yb	[µg/kg]	0.0037	0.0966	0.0212	0.7587	0.2898	0.0637	2.2761
66 Zn	[µg/kg]	1.018	26.8057	5.8931	210.5045	80.4172	17.6793	631.5136
90 Zr	[µg/kg]	0.0357	0.9393	0.2065	7.3759	2.8178	0.6195	22.1278
