# Supplementary figures and images for: Metallomic Analysis of Vitreous Humor of the Human Eye—A Post-Mortem Multielemental Study
Source: Int J Mol Sci. 2026 Mar 10;27(6):2527. doi: 10.3390/ijms27062527 (PMC13026291; doi:10.3390/ijms27062527)

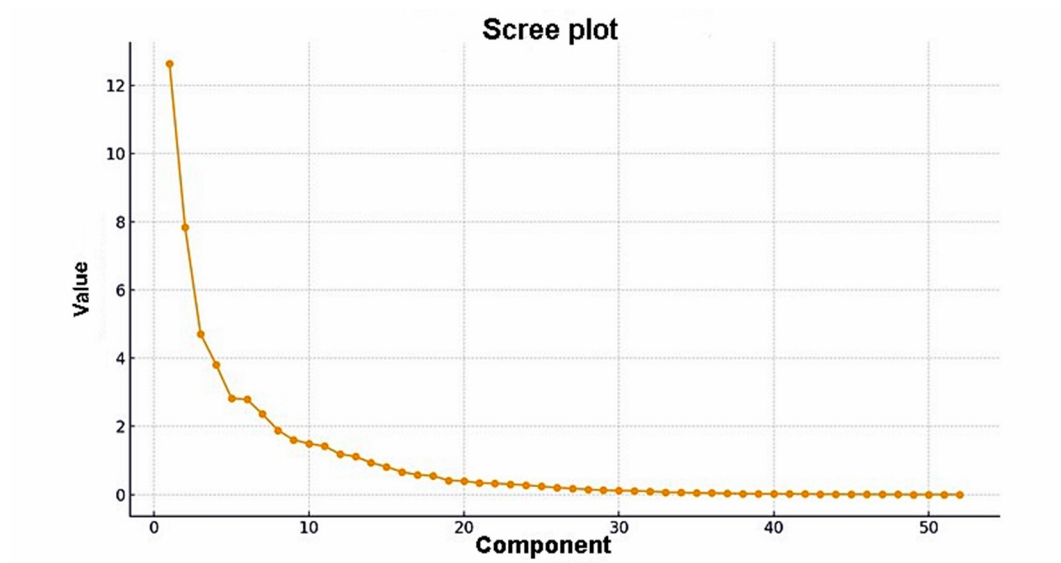

**Supplementary 4.** Scree plot of principal component analysis (PCA).

Supplement: Supplementary file 1 [file ijms-27-02527-s001.zip › Supplementary 4.pdf]
